# Application Value and Cognitive Challenges of PET/CT in the Diagnosis and Treatment of Visceral Leishmaniasis: A Retrospective Single-Center Case Series and Literature Review

**DOI:** 10.3390/diagnostics16203402

**Published:** 2026-10-19

**Authors:** Boyu Zhang, Yu Zhang, Yaqin Wu, Yue Gao, Xingxing Li, Han Wu, Yang Li, Ning Song

**Affiliations:** 1 Department of Infectious Diseases, The Second Hospital of Hebei Medical University, Shijiazhuang 050000, China; 25034101022@stu.hebmu.edu.cn (B.Z.);; 2 Department of Public Health, The Second Hospital of Hebei Medical University, Shijiazhuang 050000, China

**Keywords:** VL, PET/CT, FUO, mNGS

## Abstract

**Background and Clinical Significance:** The role of ^18^F-fluorodeoxyglucose positron emission tomography/computed tomography (PET/CT) in visceral leishmaniasis (VL) remains undefined. We sought to characterize the PET/CT features of VL and assess its diagnostic and therapeutic value. **Case Presentation:** Four VL patients undergoing PET/CT at our hospital (2022–2025) were retrospectively analyzed as a single-center case series, combined with a literature review of published VL PET/CT cases up to December 2025. Three of the four cases exhibited increased ^18^F-fluorodeoxyglucose (^18^F-FDG) uptake in the spleen, bone marrow, and lymph nodes, with additional tonsillar uptake in one case and hepatic uptake in another; one case presented with isolated lymph node uptake. In three cases, initial bone marrow smears were negative for amastigotes, and PET/CT was performed for fever of unknown origin (FUO). One case was diagnosed within one week via metagenomic next-generation sequencing (mNGS) prompted by PET/CT and epidemiological history. In the other two, VL was not suspected; they received chemotherapy/corticosteroids for presumed hemophagocytic syndrome (HPS) or connective tissue disease, and the time from symptom onset to VL diagnosis ranged from 2.5 to 4.5 months. The fourth case, confirmed by bone marrow smear, underwent PET/CT for suboptimal treatment response to exclude other diseases. Among 99 published VL cases undergoing PET/CT, indications were documented in 35, with FUO representing 83%. Initial bone marrow smears were negative in 69% of 64 evaluable cases. Of the 88 cases with evaluable PET/CT findings, 5 showed no abnormal FDG uptake. The predominant pattern was increased ^18^F-FDG uptake in the reticuloendothelial system (RES), most frequently involving the spleen (73/88, 83%), bone marrow (44/88, 50%), lymph nodes (29/88, 33%), and liver (15/88, 17%); other sites (lungs/pleura, colon, tonsils) were rarely involved. Accompanying enlargement of the spleen or liver was seen in approximately 50% of cases. The most common involvement patterns were combined splenic and bone marrow (23/88, 26%) and isolated splenic (20/88, 23%). **Conclusions:** PET/CT can provide diagnostic clues for VL, screen for comorbidities, and guide biopsy. Typical PET/CT findings—increased RES uptake without significant other organ involvement—reflect the parasite’s RES tropism. However, these findings mimic lymphoma, HPS, hematological disorders, and connective tissue diseases, risking misdiagnosis. Thus, VL should be promptly excluded in such patients using epidemiological history and etiological testing before considering alternative diagnoses.

## 1. Introduction

Visceral leishmaniasis (VL), also known as kala-azar, is a severe systemic parasitic disease caused by the protozoan parasites *Leishmania donovani* and *Leishmania infantum*, and is transmitted primarily through the bite of sandflies. The incidence of VL is influenced by multiple factors, including ecological and socioeconomic conditions, public health interventions, and surveillance capabilities, resulting in marked geographical variation worldwide. Globally, approximately 50,000 to 90,000 new cases are reported each year, leading to an estimated 20,000 to 30,000 deaths [1].

VL was historically a major epidemic in China, but after comprehensive control efforts starting in the 1950s, its incidence declined precipitously and it is now a sporadic disease restricted mainly to western provinces (e.g., Gansu, Sichuan, Shanxi, Xinjiang) [2]. Since the turn of the 21st century, although the overall incidence has remained extremely low, some historically endemic areas have shown concerning signs of resurgence. Between 2019 and 2021, a total of 608 VL cases were reported to the National Notifiable Disease Surveillance System of the Chinese Center for Disease Control and Prevention, with 158, 213, and 237 cases reported in 2019, 2020, and 2021, respectively, reflecting a steady annual increase. Currently, Hebei Province is not a core endemic area for VL; however, new resurgence hotspots have been identified there since 2020 [3].

Underdiagnosis and misdiagnosis of VL pose considerable challenges, particularly in non-endemic and impoverished regions. The clinical presentation of VL is often nonspecific, frequently manifesting as prolonged fever, and is therefore easily mistaken for common illnesses. The gold standard for diagnosis is the direct observation of *Leishmania* amastigotes (the aflagellate form) in bone marrow or spleen aspirates. The rK39 rapid diagnostic test (RDT) is a simple, rapid antibody-based test widely employed in endemic settings; however, the WHO notes that its accuracy varies by region, and it should be used in conjunction with a standardized clinical case definition rather than as an independent screening tool [4]. Moreover, it is seldom routinely implemented in non-endemic areas. Molecular diagnostic techniques, particularly polymerase chain reaction (PCR), offer exceptional sensitivity and specificity in VL diagnosis and are increasingly employed as a first-line laboratory tool, complementing clinical evaluation and conventional parasitological methods. However, the dependence of PCR on specialized equipment, reagents, and trained personnel limits its widespread application in resource-limited settings. A systematic review reported that the mean time from symptom onset to definitive diagnosis can be as long as 4.5 years, underscoring the widespread and severe nature of diagnostic delays [5].

Inadequate epidemiological awareness is a critical human factor contributing to misdiagnosis. In non-endemic areas, clinicians generally have a low index of suspicion for VL. When evaluating patients presenting with fever of unknown origin (FUO), physicians tend to focus on locally prevalent diseases and often overlook key epidemiological clues, including a history of dog exposure, travel history, immigrant background, or residence in an endemic area. This cognitive blind spot directly leads to deviations in diagnostic reasoning. Metagenomic next-generation sequencing (mNGS) and other emerging technologies have demonstrated significant potential in the etiological diagnosis of severe and refractory infections, serving as important complements to traditional methods. ^18^F-fluorodeoxyglucose positron emission tomography/computed tomography (PET/CT), as a functional imaging technique, is widely used in the etiological diagnosis of FUO. Its value lies in its ability to non-invasively and systematically localize metabolically active lesions, thereby providing critical clues for subsequent targeted diagnosis and treatment. However, the role, imaging manifestations, and diagnostic value of PET/CT in VL have rarely been reported, which may be attributable to the current low prevalence of VL.

Upon reviewing data from our hospital’s infectious disease reporting system, we observed a marked increase in VL cases from January 2022 to December 2025, compared with no cases reported before 2022 (17 vs. 0), which raised our concern. Our hospital is a Class-III Grade-A teaching general hospital located in Shijiazhuang, Hebei Province. The patients mainly originate from the surrounding areas of Shijiazhuang and neighboring regions such as Hengshui and Handan. Among these, Jingxing County and Pingshan County are not only adjacent to Shanxi—the current core epidemic area of VL—but are also historically endemic areas for VL. Therefore, we must remain highly vigilant against the resurgence of VL. In a cohort of 17 patients, 3 presented with prolonged fever as the sole clinical manifestation and underwent PET/CT scans to investigate the etiology of FUO. One patient, after being diagnosed with VL, underwent PET/CT to investigate lymphoproliferative disorders due to poor response to drug therapy. We retrospectively reviewed the PET/CT imaging characteristics and treatment processes of these 4 VL patients. Additionally, we conducted a comprehensive analysis of the literature published up to December 2025 in databases including PubMed and China National Knowledge Infrastructure (CNKI) regarding the PET/CT manifestations of VL. The aim of this study is to clarify the purposes of PET/CT examinations in VL, the PET/CT imaging features, and the application value of PET/CT in the diagnosis and treatment of VL, thereby providing evidence for clinical diagnosis and treatment of VL in non-endemic areas.

## 2. Case Presentation

### 2.1. Case Selection and Clinical Data Collection

This retrospective, single-center case series included 4 confirmed VL cases who underwent PET/CT examinations at the Second Hospital of Hebei Medical University from January 2022 to December 2025. The diagnostic criteria for VL were based on the Diagnosis and Treatment Guidelines for Visceral Leishmaniasis (2023 Edition) [6]:

(i) Suspected cases: individuals with an epidemiological history and relevant clinical manifestations;
(ii) Clinically diagnosed cases: suspected cases positive for rK39 antibody;
(iii) Confirmed cases: suspected or clinically diagnosed cases meeting any of the following criteria:
  1. Detection of *Leishmania* amastigotes on smear;
  2. Culture of *Leishmania* parasites;
  3. Positive *Leishmania* nucleic acid test;
  4. Positive *Leishmania* antigen test.

PET/CT imaging was performed on a Philips Vereos system (Philips Healthcare). Patients fasted for ≥6 h (blood glucose < 11.1 mmol/L) and received ^18^F-fluorodeoxyglucose (^18^F-FDG) at 0.15 mCi/kg (~5.5 MBq/kg) intravenously. After a 60-min uptake period, whole-body images were acquired from the supraorbital margin to the mid-femur. A CT scan (120 kVp, 318 mAs) was used for attenuation correction. PET data were acquired in 3D mode (1 min/bed, 39% bed overlap) and reconstructed using an OSEM algorithm (2 iterations, 8 subsets) with TOF and PSF modelling. Reconstruction parameters were: matrix 288 × 288, slice thickness 2 mm, display field of view 32 cm, and a Gaussian post-filter of 3 mm. Attenuation, scatter, random, and cross-calibration corrections were applied. SUVmax was calculated based on body weight and measured with a manually drawn region of interest on a dedicated workstation (Philips, version 2015).

The medical records of four patients with VL were retrieved from the electronic medical record system of the Second Hospital of Hebei Medical University. The specific data collected included: epidemiological information, clinical manifestations at presentation, laboratory tests, general imaging data, bone marrow aspiration findings, purpose of PET/CT examination, PET/CT findings, clinical interpretation and decision-making based on PET/CT results, methods of confirmation, time of confirmation, treatment approaches, and prognosis.

### 2.2. Case Series Analysis

**Figure 1:**
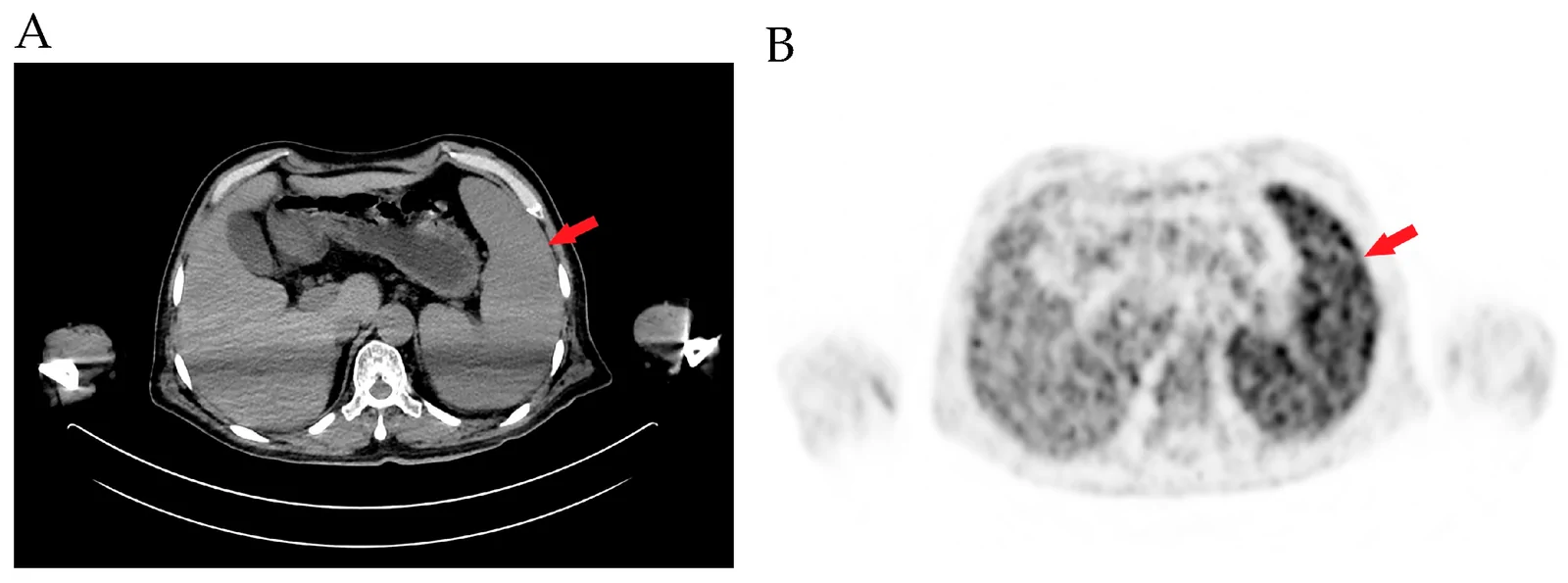

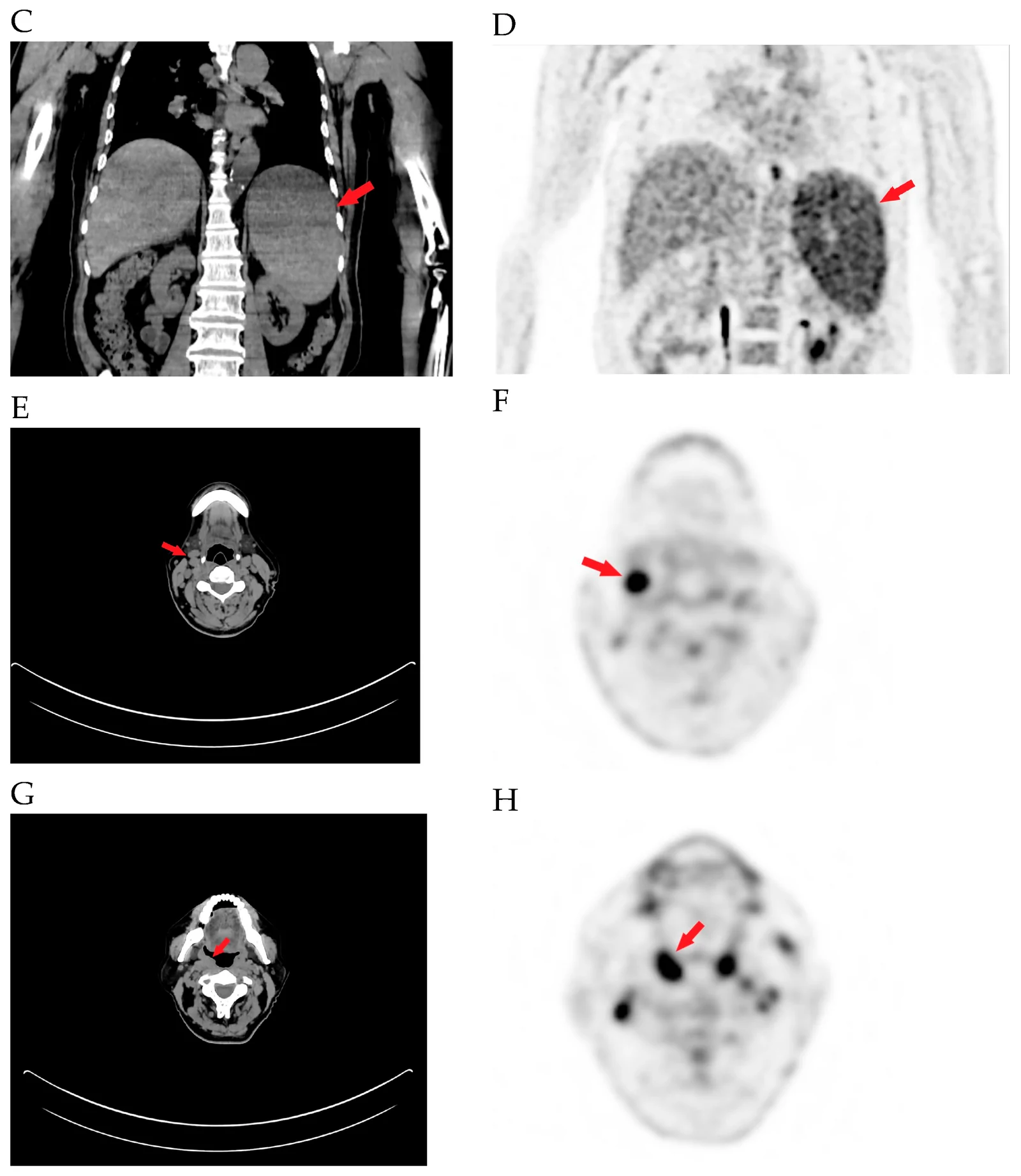
(**A**,**C**): Abdominal CT demonstrates diffuse splenomegaly without abscess formation; (**B**,**D**): PET/CT reveals splenomegaly with mildly increased metabolism. (**E**,**F**): PET/CT reveals multiple bilateral cervical lymph nodes that are slightly hypermetabolic or hypermetabolic, some slightly enlarged; (**G**,**H**): Bilateral tonsils are prominent and hypermetabolic, more pronounced on the right side. Note: (**A**,**C**,**E**,**G**) Non-contrast CT images (window level, 40 HU; window width, 250 HU). (**B**,**D**,**F**,**H**) PET images displayed in inverted grayscale with a fixed SUV window of 0–5 (black, SUV ≥ 5; white, SUV = 0). Red arrows mark the lesions. SUV, standardized uptake value.

Case 1: An elderly patient resided in a historically VL-endemic area and kept a dog at home; there was no other notable epidemiological history. The patient had a history of hypertension, diabetes, and coronary heart disease. Two weeks before presentation, the patient developed a fever without an apparent cause, with the highest temperature reaching 39.5 °C, accompanied by chills and rigors, and subsequently sought medical attention at our hospital. Abnormal laboratory findings included pancytopenia, hypoalbuminemia, hyperglobulinemia, an inverted albumin/globulin ratio, elevated C-reactive protein, hyperferritinemia, elevated erythrocyte sedimentation rate, positive antinuclear antibodies, elevated anti-centromere antibody (CENP-B IgG), and elevated IgG. Routine pathogen testing and hematological examinations revealed no abnormalities. Abdominal ultrasound and CT demonstrated hepatosplenomegaly, and lymph node ultrasound showed lymphadenopathy in the cervical, bilateral axillary, and inguinal regions (without atypical features). Microscopic examination of a bone marrow smear obtained upon admission did not reveal *Leishmania* amastigotes. Since the patient’s fever persisted despite empirical antibiotic therapy and routine investigations failed to identify its cause, PET/CT was performed. The main PET/CT findings were splenomegaly and increased uptake in the spleen (SUVmax: 4.6), bone marrow (SUVmax: 2.0–5.7), cervical lymph nodes (SUVmax: 2.3–12.8), and tonsils (SUVmax: 14.9) (Figure 1, Table 1 and Table 2). Based on the PET/CT findings, together with the patient’s residence and the dog at home, the fever was suspected to be associated with a specific infection. Peripheral blood mNGS detected *Leishmania tropica* and *Leishmania infantum*. Re-examination of the initial bone marrow smears revealed occasional *Leishmania* amastigotes, confirming the diagnosis of VL. The patient received amphotericin B cholesteryl sulfate complex and sodium stibogluconate, which led to clinical improvement and subsequent discharge.

Case 2: An elderly patient resided in a historically VL-endemic area and had no history of keeping dogs; the patient was previously healthy. Six days before presentation, the patient developed intermittent fever with a peak temperature of 39.8 °C, and subsequently sought medical attention at our hospital. Abnormal laboratory findings included pancytopenia, mildly elevated liver enzymes, elevated procalcitonin, elevated C-reactive protein, elevated erythrocyte sedimentation rate, hypoalbuminemia, hyperglobulinemia, inverted albumin/globulin ratio, hyperferritinemia, positive antinuclear antibodies, elevated anti-nucleosome antibodies, elevated anti-histone antibodies, elevated rheumatoid factor, and elevated IgG. Multiple blood cultures were negative. Peripheral blood EBV (Epstein–Barr virus)-DNA was >8000 copies/mL, and EBV lymphocyte subset analysis showed that EBV-DNA in B cells was >400 copies/1,000,000 cells, while T and NK/NKT cells were negative. Abdominal CT revealed splenomegaly, and abdominal ultrasound demonstrated hepatosplenomegaly. Microscopic examination of a bone marrow smear obtained upon admission did not reveal *Leishmania* amastigotes; bone marrow pathology and flow cytometry showed no abnormalities. Pathological examination of axillary lymph nodes revealed reactive lymphoid hyperplasia, with no immunophenotypic evidence of malignant lymphoma. Natural killer (NK) cell activity was decreased (<15.11%), while sCD25 was within the normal range. Since the patient’s fever persisted despite initial empirical antibiotic therapy and its cause remained unclear after routine investigations, PET/CT was performed 3 weeks after admission. The main PET/CT findings were several slightly hypermetabolic lymph nodes (SUVmax approximately 1.5) (Table 1 and Table 2) and splenomegaly without abnormal hypermetabolism. Empirical anti-infective therapy was ineffective. Based on the presence of fever, splenomegaly, pancytopenia, hyperferritinemia, and decreased NK cell activity, a clinical diagnosis of hemophagocytic syndrome (HPS) accompanied by EBV infection was made. Concurrent connective tissue disease could not be excluded. The patient subsequently received sodium phosphonoformate for EBV infection and the EP (etoposide and dexamethasone) plus ruxolitinib regimen, which led to fever resolution and subsequent discharge. Two months after the initial admission, the patient was readmitted with fever (peak temperature 39.9 °C). A repeat bone marrow smear still did not reveal *Leishmania* amastigotes. However, mNGS of peripheral blood detected *Leishmania infantum*, confirming HPS secondary to VL. The patient received amphotericin B cholesterol sulfate for VL and the EP regimen for HPS, which led to clinical improvement and subsequent discharge.

Case 3: An elderly patient resided in a historically VL-endemic area and had an unknown history of dog ownership; the patient had diabetes mellitus. Half a month before presentation, the patient developed intermittent fever with a peak temperature of 39.0 °C, accompanied by chills, rigors, fatigue, and night sweats, and subsequently sought medical attention at our hospital. Abnormal laboratory findings included pancytopenia, hypoalbuminemia, inverted albumin/globulin ratio, elevated C-reactive protein, hyperferritinemia, elevated IgG, positive antinuclear antibodies, elevated rheumatoid factor, and elevated anti-histone antibodies. Blood cultures were negative. Abdominal CT revealed mild hepatosplenomegaly, and lymph node ultrasound demonstrated lymph nodes in the bilateral inguinal and cervical regions. Bone marrow smear revealed hemophagocytic histiocytes, but no *Leishmania* amastigotes were found. The patient was given empirical antibiotic therapy, but the fever was not controlled. Since the cause of the fever remained unclear after routine investigations, PET/CT was performed. The main PET/CT findings were mild splenomegaly and hepatomegaly, with increased uptake in the spleen (SUVmax: 4.4–8.0), liver (SUVmax: 2.5–4.6), bone marrow (SUVmax: 1.1–3.7), and lymph nodes (SUVmax: approximately 2.0–11.2) (Table 1 and Table 2). Clinically, the differential diagnoses included HPS, bloodstream infection, and connective tissue disease. Subsequently, the patient received intravenous immunoglobulin and corticosteroids in addition to empirical antibiotics, which led to defervescence and eventual discharge. Subsequently, the patient experienced recurrent fever and was regularly treated with corticosteroids. Approximately four months after the initial admission, the patient was readmitted with high fever (peak temperature 39.6 °C, accompanied by rigors). Peripheral blood mNGS detected *Leishmania infantum*, confirming the diagnosis of VL, and the patient was transferred to a specialized infectious disease hospital for further treatment.

Case 4: An elderly patient resided in a historically VL-endemic area and had an unknown history of dog ownership; the patient had a history of autoimmune hemolytic anemia, systemic lupus erythematosus (for which regular glucocorticoid and immunosuppressant therapy was administered), and coronary heart disease. Six months prior, the patient had been hospitalized due to fever (peak temperature 39 °C) accompanied by chills, and VL was diagnosed based on the detection of *Leishmania* amastigotes on a bone marrow smear; the patient was subsequently referred to a specialized infectious disease hospital. During outpatient follow-up, abnormally elevated serum immunoglobulin levels and a positive serum immunofixation electrophoresis were detected, prompting the current admission for further evaluation. To assess the bone marrow status, a repeat bone marrow smear was performed and still revealed *Leishmania* amastigotes. Abdominal CT demonstrated splenomegaly. Given the suspicion of lymphoproliferative disorders, PET/CT was performed to guide biopsy of suspicious lesions (Table 1 and Table 2). The main PET/CT findings were increased uptake in the spleen (SUVmax: 3.2), bone marrow (SUVmax: 2.4–4.5), and lymph nodes (SUVmax: 1.6–31.4). Based on these findings, a cervical lymph node biopsy was performed, which showed reactive lymphoid hyperplasia with abundant plasma cells and histiocytes, with no immunophenotypic evidence of malignant lymphoma. Therefore, multiple myeloma, lymphoma, and other malignant hematologic diseases were essentially excluded. Considering that VL had not been cured, the patient was referred to a specialized infectious disease hospital for further treatment.

The imaging findings, clinical interpretations, and subsequent decision-making processes of the four VL cases evaluated with PET/CT are detailed in Table 1 and Table 2.

## 3. Literature Review

### 3.1. Search Strategy

A literature search was performed in PubMed, MEDLINE, Embase, Web of Science, Scopus, Google Scholar, CNKI, Wanfang Data, and the Chinese Biomedical Literature Database for articles published up to December 2025 on the PET/CT manifestations of VL. The search strategy was as follows: (“Leishmaniasis, Visceral” OR “visceral leishman*”OR “Kala-azar” OR “Kala azar”) AND (“Positron-Emission Tomography” OR “Tomography, Emission-Computed” OR “PET” OR “PET-CT” OR “PET/CT” OR “PET CT” OR “FDG-PET” OR “FDG PET” OR “FDG-PET/CT” OR “Fluorodeoxyglucose F18” OR “FDG”). Additionally, supplementary records were identified by screening the reference lists of the included articles.

We considered all available full-text articles and conference abstracts that reported on the application of PET/CT in human patients with VL. The same eligibility criteria were applied uniformly to both document types:

1. a diagnosis of VL that fulfilled the criteria for definitive diagnosis or clinical diagnosis specified in this review (see “definitions of diagnosis” below);
2. the article contained original patient data;
3. sufficient clinical details and PET/CT imaging findings were provided.

To avoid double counting of patients, we excluded duplicate papers and, for eligible articles that made reference to previously reported cases, extracted only the newly described original cases. When a conference abstract and a full-text article described overlapping cases, the full-text article served as the primary source; the abstract was retained only if it provided unique, otherwise unavailable data. All retained studies then underwent a systematic deduplication process. Two reviewers (BZ and YZ) independently screened titles, abstracts, and full-text articles for eligibility; disagreements were resolved through discussion with a third reviewer (NS). To further detect any remaining overlapping cases, all authors collaboratively reviewed each included study, recording authors’ names, institutional affiliations, recruitment period, geographic setting, and patient-level characteristics (age, sex, clinical history, and imaging descriptions). When studies exhibited overlapping authorship, similar recruitment periods, or common geographic settings, a side-by-side comparison of the reported patients was performed. Any duplicated or previously reported cases were counted only once. After this process, the entire dataset was reassessed using the same institutional and patient-level criteria, and no further duplicates were found. Uncertainties during deduplication were resolved through discussion among all co-authors.

Owing to differences in VL endemicity and diagnostic resources, the same diagnostic criteria were not applied to the local and literature cohorts. Therefore, for the purpose of this literature review, “definitive diagnosis” was defined as the presence of direct parasitological or molecular evidence of *Leishmania* infection, including: identification of amastigotes in tissue smears or histopathological specimens (e.g., bone marrow, spleen, liver, lymph node, colonic tissue); a positive result by PCR or other nucleic acid amplification test; a positive culture; or detection of *Leishmania* nucleic acids by mNGS. In contrast, “clinical diagnosis” was defined as a diagnosis of VL based on consistent clinical and epidemiological findings in the presence of a positive serological test for anti-*Leishmania* antibodies, or when the original article documented a clinical diagnosis of VL without reporting a specific confirmatory method. Cases with any direct parasitological or molecular evidence were classified as definitive diagnoses; cases meeting only the clinical or serological criteria, or without a reported confirmatory method, were classified as clinical diagnoses.

The following information was extracted and comprehensively analyzed: publication year; patients’ geographic regions of medical consultation; age; sex; underlying diseases; immunosuppression status; indication for PET/CT; initial bone marrow smear/biopsy findings; general imaging and physical examination findings for hepatomegaly, splenomegaly, and lymphadenopathy; PET/CT findings; diagnosis after PET/CT; time from PET/CT to VL confirmation; and process and methods for VL confirmation after PET/CT.

For the cases identified through the literature review, PET/CT findings were classified into three categories based strictly on the explicit terminology used in the original articles:

1. Scans with explicitly reported abnormal FDG uptake (Explicitly Positive): The original text must contain explicit descriptors of increased metabolism, rather than solely CT morphology.
2. Scans with explicitly reported absence of abnormal FDG uptake (Explicitly Negative): The report clearly states a negative finding.
3. Scans with non-evaluable FDG uptake status (Non-evaluable): PET/CT scans that cannot assess VL-related organ involvement are classified as non-evaluable. This includes: FDG uptake status being entirely abnormal, suspicion of visceral involvement being based purely on CT morphological descriptions without any explicit metabolic descriptor, or abnormal FDG uptake reported being confined to sites incompatible with VL pathophysiology.

For each included case, an organ was recorded as positive only when the original report explicitly described abnormal/increased FDG uptake in that organ. A PET/CT scan was considered positive if it showed such explicitly described uptake in ≥1 organ.

Furthermore, patients were categorized into an immunocompetent group and an immunocompromised group to evaluate differences in PET/CT findings between the two groups. Immunocompromised status was defined as meeting any of the following: use of corticosteroids or immunosuppressants at the time of PET/CT, receipt of radiotherapy or chemotherapy for malignancy, HIV co-infection, or an immunocompromised state documented in the literature.

### 3.2. Main Findings

The literature review aimed to comprehensively evaluate the current role, diagnostic value, and challenges of PET/CT in VL, and to systematically summarize the clinically relevant imaging findings. The literature search identified 7594 records across nine databases (Google Scholar, PubMed, Medline, CNKI, Scopus, Chinese Biomedical Literature Database, Web of Science, Wanfang Data, and Embase). After deduplication and title/abstract screening, 7529 records were excluded, leaving 65 full-text articles for eligibility assessment. An additional 5 records were identified through reference list screening; no prospective studies or clinical trials were identified. All 70 articles involving a total of 99 VL cases who underwent PET/CT examinations fulfilled the inclusion criteria and were ultimately included in the analysis. Table S1 (provided in the Supplementary Material) summarizes the data extracted from the selected articles [7,8,9,10,11,12,13,14,15,16,17,18,19,20,21,22,23,24,25,26,27,28,29,30,31,32,33,34,35,36,37,38,39,40,41,42,43,44,45,46,47,48,49,50,51,52,53,54,55,56,57,58,59,60,61,62,63,64,65,66,67,68,69,70,71,72,73,74,75,76,77].

During the literature review, a potential case overlap was identified between the studies by Morigi et al. [63] and Zanoni et al. [44]. Both studies included a common author from the same institution, and the enrollment periods overlapped (Zanoni et al. [44]: 2008–2017; Morigi et al. [63]: 2008–January 2014). To avoid double-counting of patients, the 10 cases reported by Morigi et al. [63] were excluded from the analysis, and only the cases from Zanoni et al. [44] were retained. All screening totals, denominators, and case counts have been updated accordingly. The reference is to Morigi et al. [63] is cited here but should be identified as an excluded duplicate. For the study by Zanoni et al. [44], we observed that the sum of individually listed PET-positive cases in the organ involvement table equalled 23, whereas the text repeatedly stated that 21 of the 23 patients had positive PET/CT (with the 2 immunocompromised patients explicitly described as scan-negative). Because the origin of this numerical difference could not be resolved in a secondary analysis, we adopted the following usage: (i) for organ distribution, combination pattern, and other analyses, all 23 positive records from the organ involvement table and the 2 negative patients (immunosuppressed, negative scans) were retained to make full use of the published anatomical information; (ii) for the immune-status subgroup analysis, only those patients whose immune status was unequivocally linked to their scan results were included—i.e., 21 immunocompetent (positive) and 2 immunosuppressed (negative). The two additional positive records from the organ involvement table in that study (which listed all PET/CT-positive cases) were not assigned to any immune-status category. This approach is transparently noted in the relevant tables and figures, and its nature as an inherent limitation of literature-based reviews is mentioned in the Limitations section.

**Figure 2:**
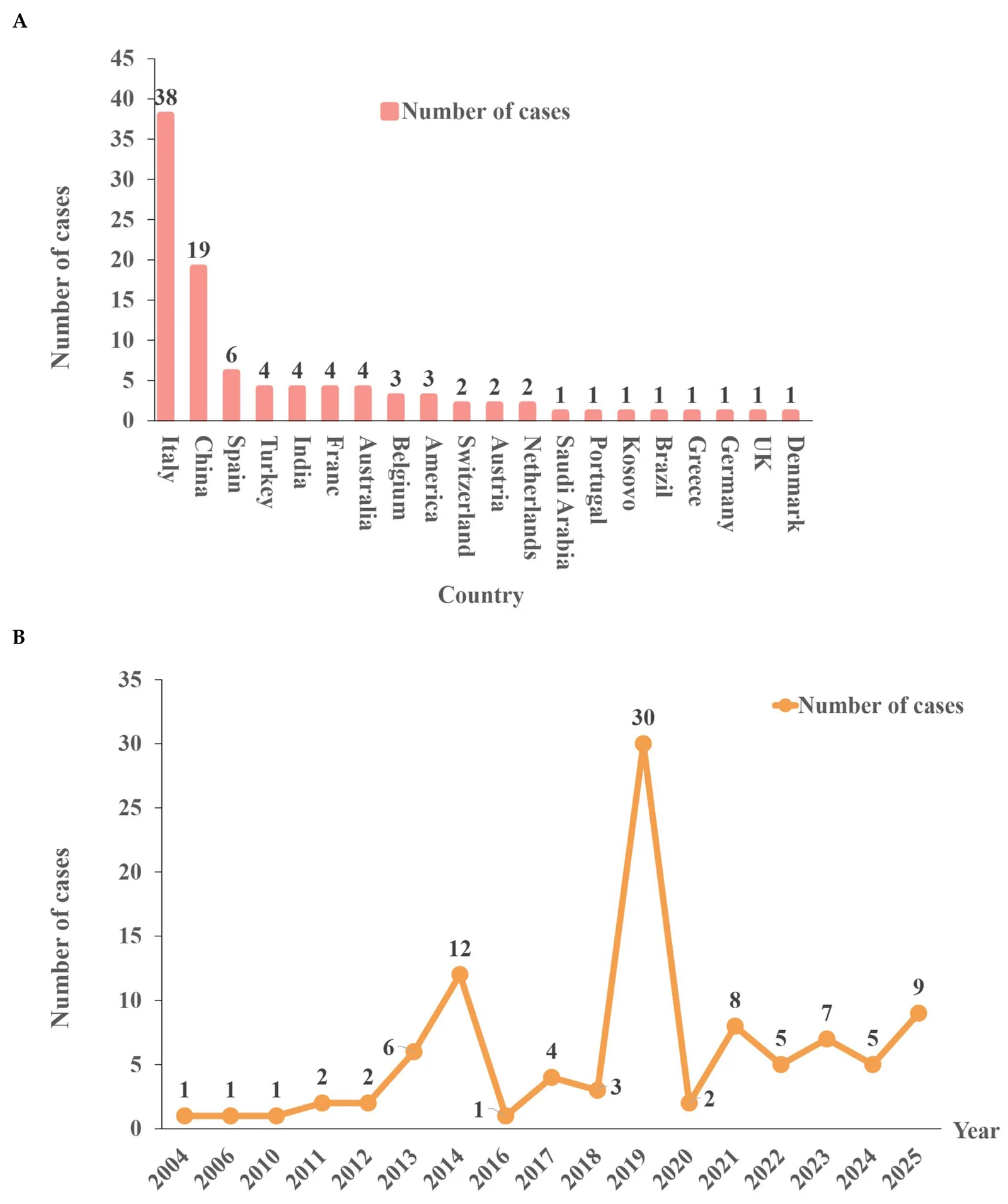
(**A**): Distribution of the number of VL cases undergoing PET/CT by country. (**B**): Distribution of the number of VL cases undergoing PET/CT by year.

**Figure 3:**
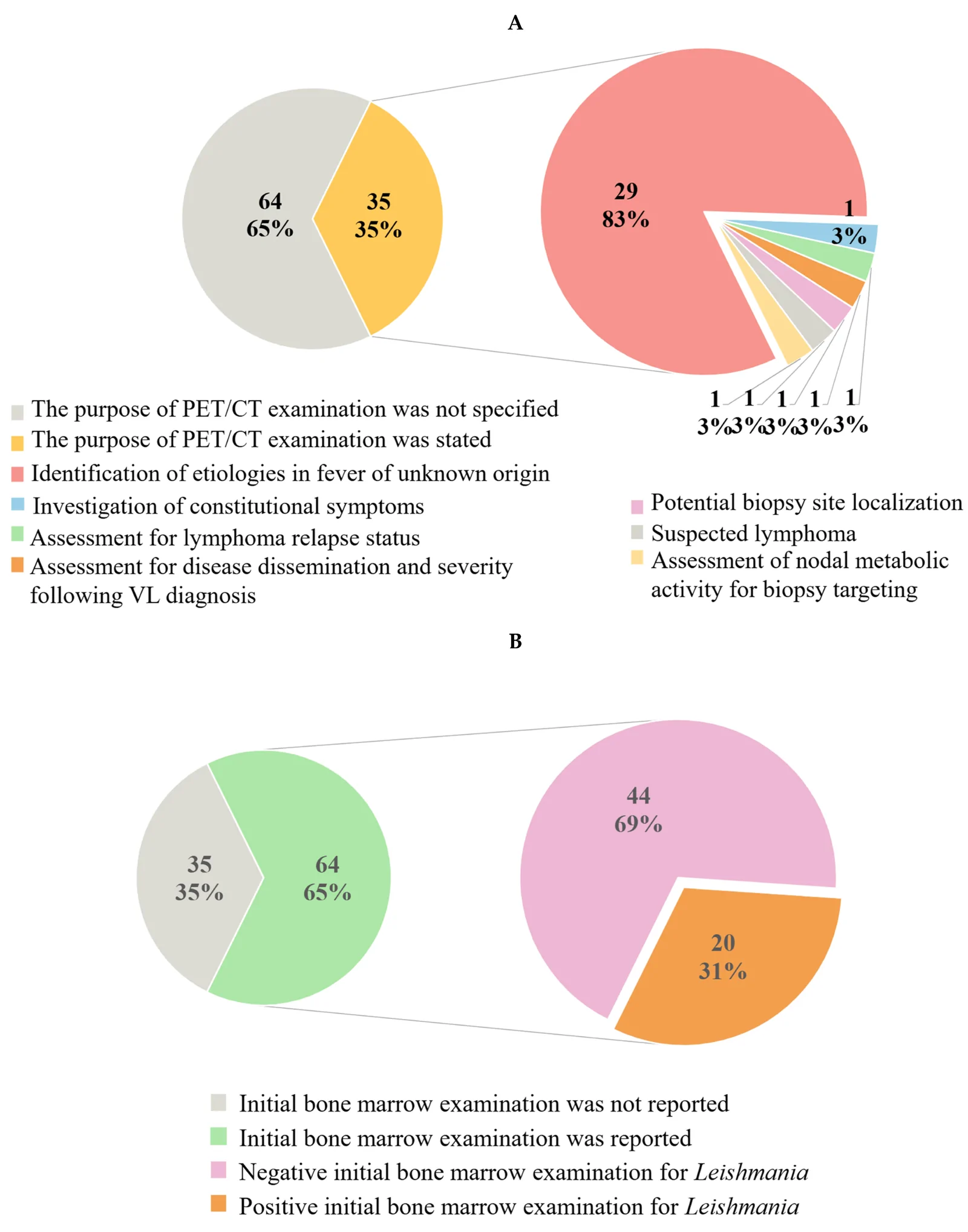

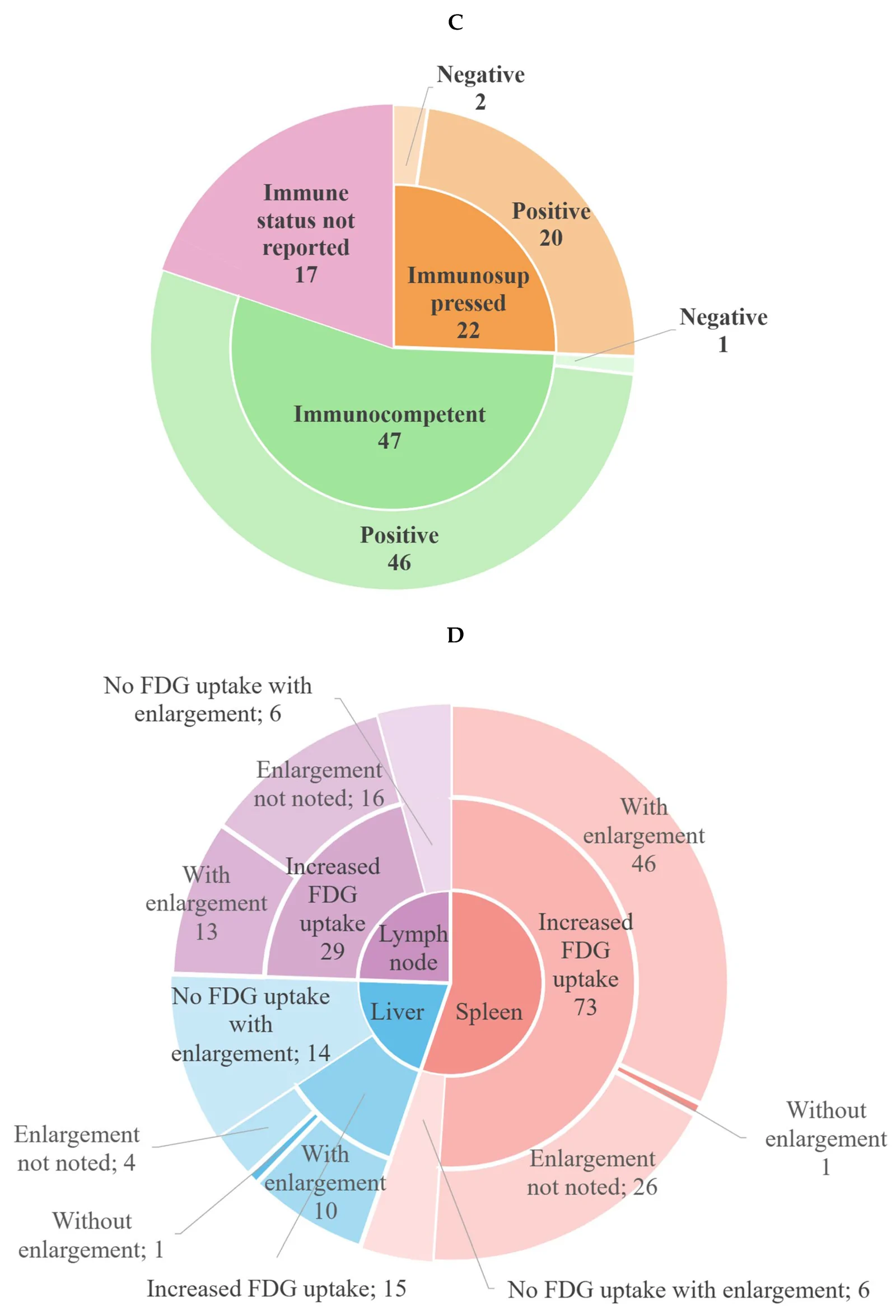

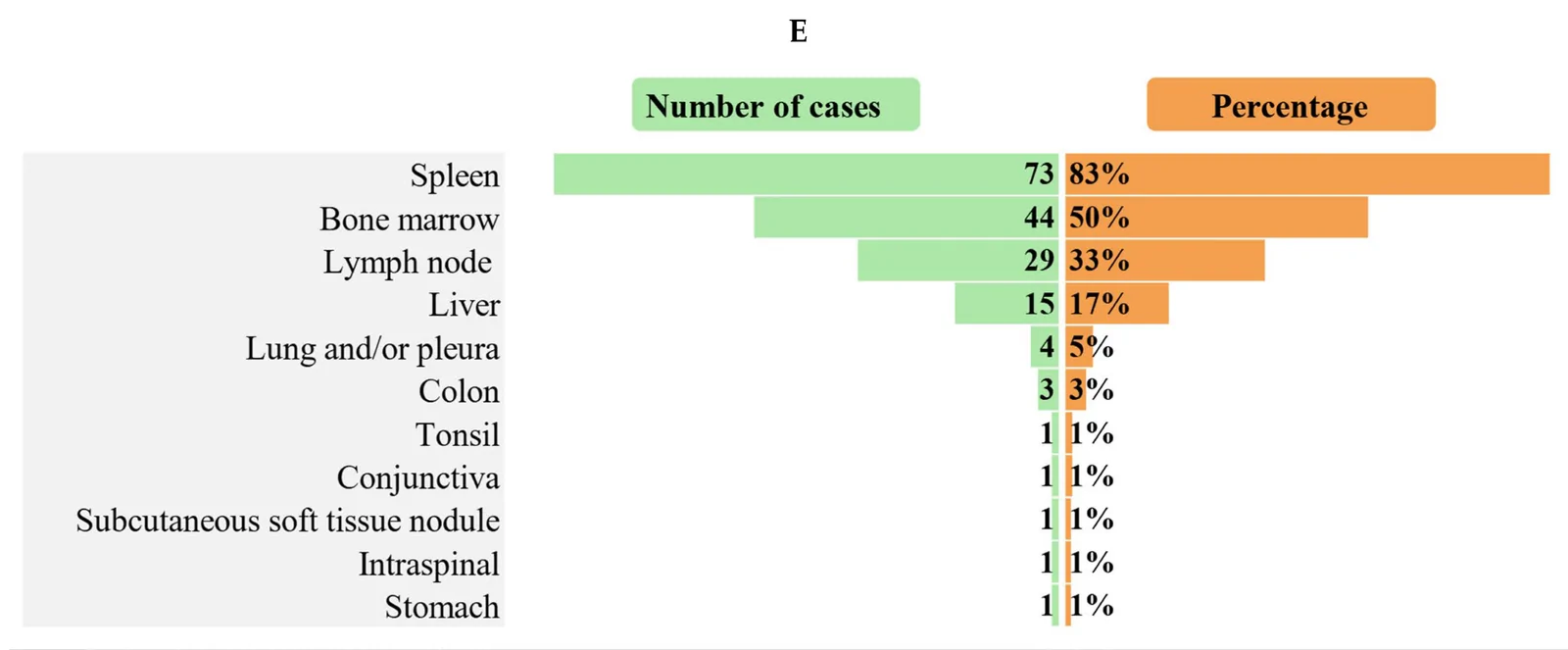
(**A**): Purpose of PET/CT examination in VL cases. Note: In 35 of 99 cases (35/99, 35%), the purpose of PET/CT was explicitly documented. The indications included: identification of etiologies in fever of unknown origin (29/35, 83%), investigation of constitutional symptoms (1/35, 3%), assessment for lymphoma relapse (1/35, 3%), assessment for disease dissemination and severity following VL diagnosis (1/35, 3%), potential biopsy site localization (1/35, 3%), suspected lymphoma (1/35, 3%), and assessment of nodal metabolic activity for biopsy targeting (1/35, 3%). (**B**): Positive rate of initial bone marrow microscopy in VL cases undergoing PET/CT. Note: In 64 of 99 cases (64/99, 65%), an initial bone marrow examination result was explicitly reported. Among these, 44/64 (69%) were negative for *Leishmania*, while 20/64 (31%) were positive. (**C**): Distribution of positive and negative PET/CT findings by immune status. Note: This section was performed excluding the 11 non-evaluable cases. Figure 3C shows the proportions of explicitly positive and explicitly negative PET/CT scans among immunocompetent and immunocompromised cases; numbers represent the number of cases. Immunosuppression status for each case is listed in Table S1. No formal statistical comparison was performed between the groups, and the numerical difference should not be interpreted as evidence of a true difference in diagnostic yield. In the immune-status subgroup analysis of Zanoni et al. [44], only patients whose immune status was unequivocally linked to their scan results were included—21 immunocompetent (positive) and 2 immunosuppressed (negative). Two additional positive records from the summary table of PET/CT-positive cases in that study lacked immune status assignment and were therefore excluded; see the Main Findings section for handling of the numerical difference. (**D**): Reported frequency of splenomegaly, hepatomegaly, and lymphadenopathy in compiled VL cases. Note: This section was performed excluding the 11 non-evaluable cases. Numbers in the figure represent the number of cases. (**E**): Distribution of organs showing increased FDG uptake among reviewed VL cases. Note: This section was performed excluding the 11 non-evaluable cases. Numbers in the figure represent the number of cases. Of the 88 cases, organ involvement was distributed as follows: spleen in 73/88 (83%), bone marrow in 44/88 (50%), lymph nodes in 29/88 (33%), liver in 15/88 (17%), lung and/or pleura in 4/88 (5%), colon in 3/88 (3%), tonsil in 1/88 (1%), conjunctiva in 1/88 (1%), subcutaneous soft tissue nodule in 1/88 (1%), intraspinal region in 1/88 (1%), and stomach in 1/88 (1%). For the study by Zanoni et al. [44], 23 positive cases were extracted from the detailed organ involvement table, whereas the authors’ text reported a total of 21 positive patients. To preserve all reported anatomical information, the 23 cases were retained; this does not materially affect the relative organ frequencies (see the Main Findings section for details).

Among the reported cases, Italy had the highest number of cases, followed by China (Figure 2A), and the annual number of case reports showed an upward trend (Figure 2B). Of the 99 cases, 35 explicitly stated the purpose of the PET/CT examination, most commonly for the differential diagnosis of FUO (29/35 cases, 83%; Figure 3A). Other purposes included guiding biopsy (2 cases), investigation of constitutional symptoms (1 case), assessment of lymphoma relapse status (1 case), evaluation for suspected lymphoma (1 case), and evaluation of disease extent and severity after VL diagnosis (1 case). Of the 99 cases, 64 had documented initial bone marrow examination results; among these, 44 (44/64, 69%) showed no evidence of *Leishmania* amastigotes on initial bone marrow examination (Figure 3B).

Based on the explicit terminology (FDG uptake description) used in the original articles, the cases obtained from the literature review were classified into three categories: Explicitly Positive (*n* = 83), Explicitly Negative (*n* = 5), and Non-evaluable (*n* = 11) (Table 3). In the subsequent analyses, the three sections “Distribution of positive and negative PET/CT findings by immune status,” “Reported frequency of splenomegaly, hepatomegaly, and lymphadenopathy,” and “Distribution of organs showing increased FDG uptake” were performed excluding the 11 non-evaluable cases. The remaining analyses were based on the entire set of 99 cases identified through the literature review.

Among the immunocompetent patients collated in this literature review, PET/CT demonstrated abnormal findings in 46 of 47 individuals; among immunosuppressed patients, such findings were documented in 20 of 22. As a reference for variability, exact 95% confidence intervals (Clopper–Pearson method) for the observed positivity proportions were calculated. For the immunocompetent group, the interval was 88.7–99.9%; for the immunosuppressed group, it was 70.8–98.9%. Since the data originate from individual published reports and the group sizes are unbalanced, no statistical comparison of positivity proportions was undertaken, and the numerical difference should not be extrapolated to imply a difference in clinical diagnostic performance. (Figure 3C).

The most common PET/CT findings in VL were increased FDG uptake in the spleen (73/88, 83%), bone marrow (44/88, 50%), lymph nodes (29/88, 33%), and liver (15/88, 17%). Increased uptake in the lungs/pleura (4/88, 5%) and colon (3/88, 3%) was less frequent. Increased FDG uptake has also been reported in the tonsils, conjunctiva, subcutaneous soft tissue nodules, intraspinal regions, and stomach (Figure 3E). Among the 88 patients, 46 presented with splenomegaly and increased splenic FDG uptake, 10 presented with hepatomegaly and increased hepatic FDG uptake, and 13 presented with lymphadenopathy and increased FDG uptake in the lymph nodes (Figure 3D). Additionally, 1 presented with increased splenic FDG uptake and without splenomegaly, 1 presented with increased hepatic FDG uptake and without hepatomegaly, 6 presented with isolated splenomegaly without increased FDG uptake, 14 with isolated hepatomegaly without increased FDG uptake, and 6 with isolated lymphadenopathy without increased FDG uptake (Figure 3D).

Among the patterns of organ involvement with abnormal FDG uptake (Table 4), involvement of the spleen and bone marrow was the most common (23/88, 26%), followed by concurrent isolated splenic involvement (20/88, 23%) and involvement of the spleen, bone marrow, and lymph nodes (8/88, 9%). Other patterns were relatively less frequent (Table 4). Of the 73 cases exhibiting increased splenic FDG uptake, 29 (40%) showed diffuse uptake, 13 (18%) showed diffuse uptake with superimposed focal uptake, and 2 (3%) showed isolated focal uptake. The specific patterns of splenic uptake in the remaining cases were not described.

**Figure 4:**
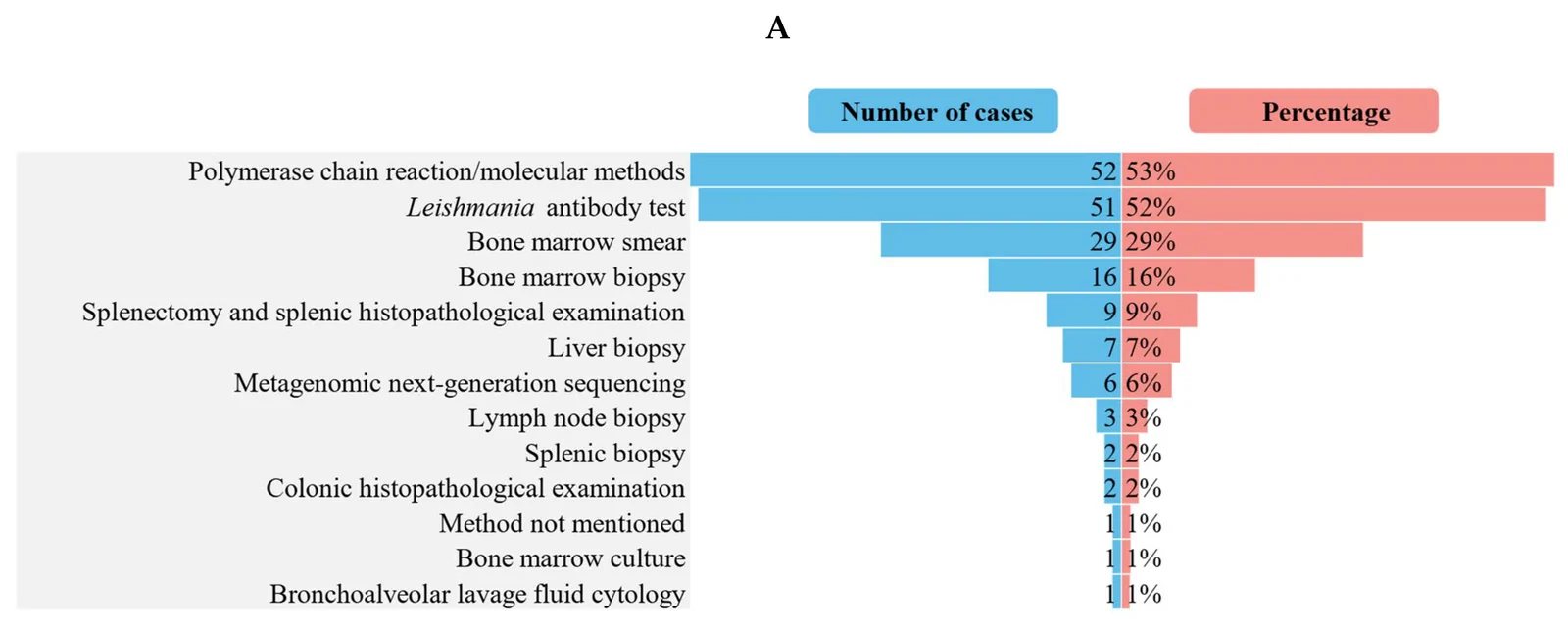

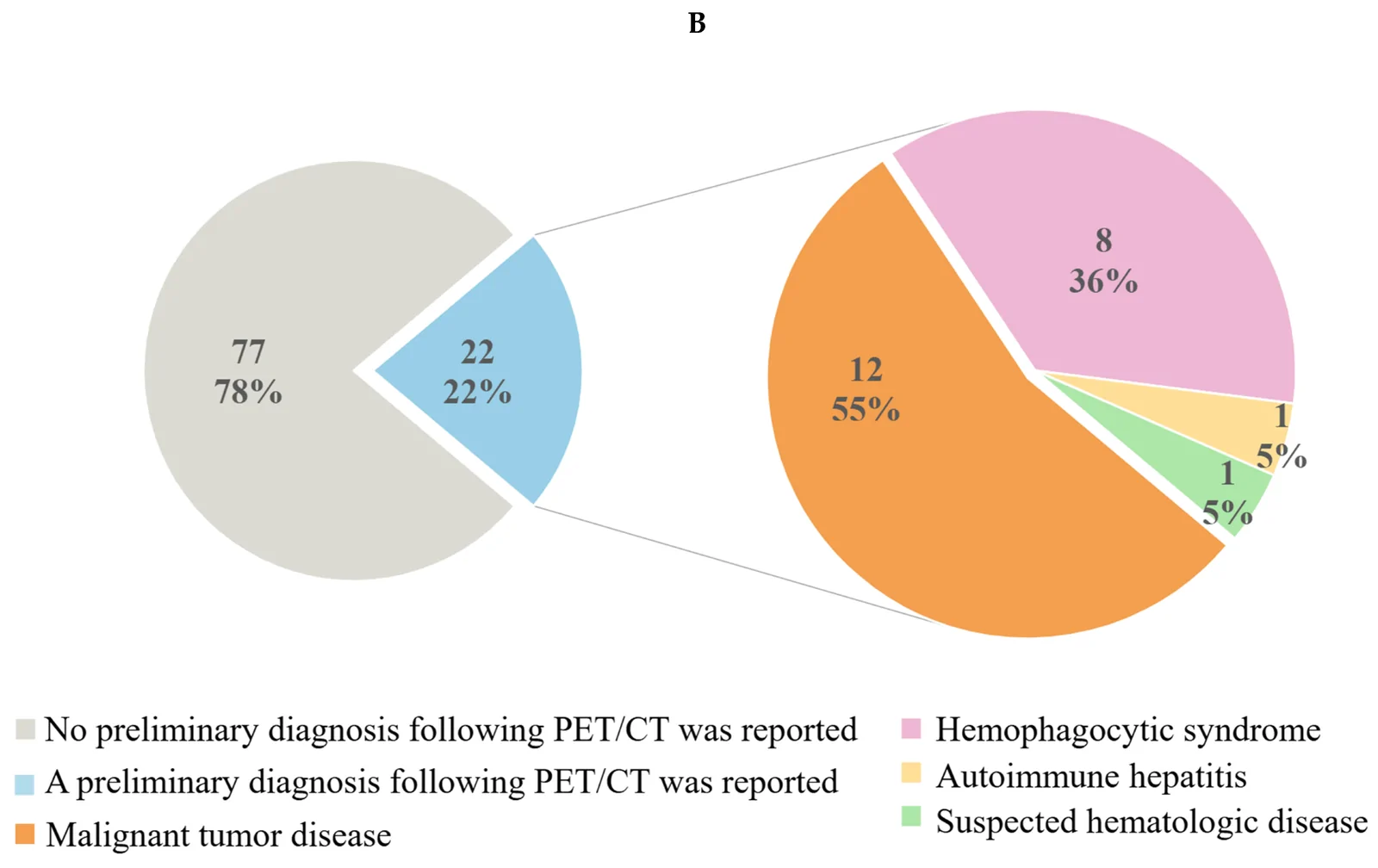
(**A**): Reported diagnostic methods for VL after PET/CT in the 99 included cases. Note: VL was diagnosed in all 99 cases after PET/CT. Of these, 93 were classified as definitive diagnoses on the basis of direct parasitological or molecular evidence, and 6 were classified as clinical diagnoses (5 with serological evidence alone and 1 with no specific confirmatory method reported). Because some cases were confirmed by more than one modality, the diagnostic categories are not mutually exclusive. The diagnostic methods used were as follows: PCR/molecular methods (52/99, 53%), *Leishmania* antibody test (51/99, 52%), bone marrow smear (29/99, 29%), bone marrow biopsy (16/99, 16%), splenectomy and splenic histopathological examination (9/99, 9%), liver biopsy (7/99, 7%), mNGS (6/99, 6%), lymph node biopsy (3/99, 3%), splenic biopsy (2/99, 2%), colonic histopathological examination (2/99, 2%), bone marrow culture (1/99, 1%), bronchoalveolar lavage fluid cytology (1/99, 1%), and method not mentioned (1/99, 1%). The cases reported by Zanoni et al. [44] were diagnosed by serological and molecular methods, as stated in the original article, and are therefore counted in both the “Leishmania antibody test” and “PCR/molecular methods” categories. The 1 case classified as “Method not mentioned” had a clinical diagnosis of VL documented in the original article, although the specific confirmatory method was not detailed. (**B**): Preliminary diagnosis after PET/CT in VL cases. Note: In 22 of 99 cases (22%), a preliminary diagnosis following PET/CT was explicitly reported. These included malignant tumor disease (12/22, 55%), hemophagocytic syndrome (8/22, 36%), autoimmune hepatitis (1/22, 5%), and suspected hematologic disease (1/22, 5%).

Notably, although PET/CT may provide helpful diagnostic clues, the definitive diagnosis is usually established through other techniques. VL was diagnosed in all 99 cases after PET/CT. The diagnostic methods used (with some cases confirmed by more than one modality) were as follows (Figure 4A): PCR/molecular methods (52/99, 53%), Leishmania antibody test (51/99, 52%), bone marrow smear (29/99, 29%), bone marrow biopsy (16/99, 16%), splenectomy and splenic histopathological examination (9/99, 9%), liver biopsy (7/99, 7%), mNGS (6/99, 6%), lymph node biopsy (3/99, 3%), splenic biopsy (2/99, 2%), colonic histopathological examination (2/99, 2%), bone marrow culture (1/99, 1%), bronchoalveolar lavage fluid cytology (1/99, 1%), and method not mentioned (1/99, 1%). Of these, 93 cases were confirmed by direct parasitological or molecular evidence (definitive diagnosis), while 5 cases were diagnosed based on serology alone and 1 case had no specific confirmatory method reported; the latter 6 cases were therefore classified as clinical diagnoses.

Of the 99 cases, 22 had a documented initial diagnosis following PET/CT examination. The reported provisional diagnoses included lymphoma and other malignant neoplasms (12 cases), HPS (8 cases), a hematological disorder (1 case), and autoimmune liver disease (1 case) (Figure 4B).

## 4. Discussion

To date, PET/CT examinations for VL have been rarely reported, and large-sample case series are virtually nonexistent. This may be attributable to the effective global control of VL, historically a widespread infectious disease, which has led to a declining number of cases. Our institution had no reported VL cases before 2022; however, 17 cases were reported in the four years after 2022, suggesting the presence of re-emergence of sporadic VL cases in historically endemic areas—a phenomenon that warrants attention. In recent years, case reports of VL patients undergoing PET/CT examinations have shown a steady increasing trend, with a notable number originating from Italy and China. This reflects growing attention to the application of PET/CT in the diagnosis and treatment of VL. However, in resource-limited regions, the use of PET/CT is somewhat limited by issues of accessibility and the relatively high cost. The purposes of PET/CT examination in VL, the typical PET/CT findings, and the actual diagnostic and therapeutic value of PET/CT in VL management are all topics that warrant further investigation.

In this study, of the 4 VL cases, 3 underwent PET/CT prior to diagnosis. All three patients had bone marrow smear examinations, yet no *Leishmania* amastigotes were detected. In these three patients, the purpose of PET/CT was to determine the etiology of FUO. Among published cases that documented the purpose of PET/CT examination, 83% underwent the procedure to determine the etiology of FUO. PET/CT is commonly used in the etiological workup of FUO, helping to identify infectious foci, screen for occult tumors and hematologic diseases, and detect connective tissue diseases such as Takayasu arteritis. Conventional imaging in VL (e.g., ultrasound or CT) primarily provides morphological information. In contrast, FDG PET/CT can provide functional metabolic data and has the theoretical potential to detect pathological activity before morphological changes become evident [7]; however, this temporal precedence has not been specifically validated in VL and warrants prospective study.

The fourth case involved a patient who had been diagnosed with VL six months earlier and had received treatment, but the therapeutic effect was unsatisfactory. A repeat bone marrow smear revealed the persistent presence of *Leishmania* amastigotes. To rule out lymphoproliferative disorders, the patient underwent a PET/CT examination to guide lymph node biopsy, which ultimately excluded diseases such as lymphoma. This suggests that PET/CT not only aids in the differential diagnosis of FUO etiology, but also has value in assessing comorbidities and guiding targeted biopsies—a finding substantiated by the literature review. VL can occur in association with other systemic diseases involving the reticuloendothelial system (RES), and may also develop secondary to such conditions. PET/CT is of value in identifying these comorbidities. Banegas et al. reported a case in which a patient with lymphoma in remission underwent follow-up PET/CT and was found to have newly developed homogeneous splenomegaly with increased uptake, ultimately diagnosed as VL [18]. Dunzinger et al. reported a case of a melanoma patient who presented with newly developed focal areas of increased hepatic uptake on PET/CT, which were difficult to differentiate from the primary tumor; VL was confirmed by iliac crest biopsy and peripheral blood PCR, and follow-up PET/CT after anti-leishmanial treatment showed disappearance of the lesions [32]. P. Tatarelli reported a case of Hodgkin lymphoma in which newly increased uptake was noted in the spleen, bone marrow, and lymph nodes; *Leishmania* amastigotes were found on bone marrow aspiration, and the PET/CT lesions resolved after anti-leishmanial treatment [47]. Gilles Darcis reported a case of Hodgkin lymphoma with rapidly progressive lymph node lesions and increased FDG uptake; lymph node biopsy revealed abundant *Leishmania* amastigotes and the lesions regressed after anti-leishmanial therapy [50]. Therefore, when PET/CT reveals new RES involvement in patients with tumors such as lymphoma, VL should also be suspected.

In this cohort, none of the four patients underwent follow-up PET/CT, possibly due in part to the high cost of the procedure. Among previously reported cases, a total of eight cases underwent follow-up PET/CT after effective anti-leishmanial treatment, and all showed a reduction or complete resolution of previously hypermetabolic lesions (Table S1). These preliminary observations, based on a limited number of published case reports, suggest that FDG-PET/CT may be a promising imaging modality for monitoring treatment response and detecting relapse or progression in visceral leishmaniasis. However, given the small sample size and inherent selection bias, these findings are insufficient to establish PET/CT as a validated tool for this purpose, and larger prospective studies are needed to confirm its clinical utility [9,16,32,33,47,50,54,68].

Overall, on PET/CT, all four cases demonstrated increased lymph node uptake. Increased splenic uptake was observed in three cases (Cases 1, 3, and 4); increased bone marrow uptake in three (Cases 1, 3, and 4); increased hepatic uptake in one (Case 3, accompanied by a slightly prominent liver); and increased tonsillar uptake in one (Case 1). With regard to morphological abnormalities, splenomegaly was present in all four patients, a slightly prominent liver was noted exclusively in Case 3, and lymphadenopathy—defined as lymph node enlargement or cortical thickening on imaging—was identified in three patients (Cases 1, 3, and 4; slightly enlarged nodes in Case 1, mild cortical thickening in Case 3, and enlarged nodes in Case 4). A review of the literature revealed that 73(73/88, 83%) of cases exhibited increased FDG uptake in the spleen, and 46 cases had concomitant splenomegaly; 50% showed increased bone marrow uptake; 15(15/88, 17%) exhibited increased hepatic FDG uptake, and 10 cases had concomitant hepatomegaly; 29(33%) demonstrated lymph node FDG uptake, and 13 cases had concomitant lymphadenopathy. Therefore, increased FDG uptake in RES organs, such as the spleen, bone marrow, and lymph nodes, with or without splenomegaly, hepatomegaly, or lymphadenopathy, represents a relatively typical PET/CT manifestation of VL. This finding is consistent with the core pathological feature of VL—diffuse and reactive mononuclear phagocyte proliferation resulting from parasitic infiltration of the RES. Some cases did not exhibit splenomegaly, hepatomegaly, or lymphadenopathy but showed increased FDG uptake, suggesting that PET/CT has the theoretical potential to detect metabolic changes before significant structural alterations occur. However, this temporal precedence has not been specifically validated in VL and warrants prospective investigation. Therefore, PET/CT may serve as a valuable complement to conventional imaging. Additionally, conventional imaging (including ultrasound and CT) revealed hepatomegaly in three patients (Cases 1–3), whereas PET/CT showed no notable liver abnormality except in Case 3, who exhibited a slightly prominent liver with mildly increased metabolic activity. This discrepancy may reflect inherent differences between structural and metabolic imaging modalities in terms of measurement criteria, resolution, and operator-dependent factors. Immunocompromised status (particularly cellular immune deficiency) not only significantly increases the risk of VL but may also lead to a diminished inflammatory response, reduced local metabolic rate in affected organs, and atypical PET/CT findings. In this study, PET/CT demonstrated abnormalities in most patients regardless of immune status. Because the findings are compiled exclusively from small, unbalanced case series, formal comparative analysis was not feasible, and the observations should be interpreted solely as descriptive data. It is hypothesized that although the inflammatory response in affected organs is weaker in immunocompromised hosts, the extensive proliferation and direct infiltration of parasites in the RES, together with their inherently active metabolism, may result in high FDG uptake. In contrast, immunocompetent hosts primarily limit the pathogen through inflammatory responses and immune activation, which also manifests as hypermetabolism. Diffuse splenic FDG uptake is the most common PET/CT manifestation of VL [25,33,44]. Of note, VL may also present as multiple focal, solitary, pseudonodular areas of heterogeneous FDG hypermetabolism in the spleen, which can easily be misdiagnosed as splenic lymphoma or metastatic tumor [43,75]. Recognizing this atypical manifestation is crucial for avoiding the misdiagnosis of VL as a malignant tumor and thereby preventing unnecessary splenectomy or chemotherapy. Increased FDG uptake in the spleen may be associated with nodular hyperplasia of the red pulp and macrophage activation caused by *Leishmania* infection, and its pattern is pathologically suggestive: focal uptake often reflects local inflammation or granuloma formation, whereas diffuse uptake is primarily related to systemic infection and splenic congestion [44,64].

Zanoni et al. [44] reported that in PET/CT-positive VL cases, 65% exhibited extrasplenic FDG hypermetabolic lesions, with bone marrow involvement being the most common (average SUVmax of approximately 4.0), followed by lymph node involvement, and occasional pulmonary involvement. Diffuse FDG uptake in the bone marrow is another significant feature of VL, reflecting involvement of the hematopoietic system and enhanced activity of bone marrow macrophages, consistent with the clinical presentation of pancytopenia [25,33,44]. Zanoni et al. [44] also noted that enhanced FDG uptake in the bone marrow may indicate a higher rate of parasite replication and a greater likelihood of detecting *Leishmania* parasites in bone marrow biopsies. FDG uptake in lymph nodes and the liver can occur independently or concurrently with splenic and bone marrow lesions, suggesting possible direct invasion by *Leishmania* parasites and induction of diffuse macrophage activation. FDG uptake in the liver may also present as either a diffuse or a focal pattern. Lymph node involvement can encompass multiple sites, including the cervical, abdominal, mediastinal, and inguinal lymph nodes. These findings further substantiate that VL is a systemic disease involving the RES, and PET/CT can directly visualize its involvement across various body regions. Although both the spleen and liver are components of the RES, increased FDG uptake in the spleen was far more frequently reported than in the liver among the reviewed VL cases. Given that the spleen is a major site of parasite sequestration and tissue remodeling in VL, it is plausible that higher FDG uptake reflects a greater parasite burden, more severe structural disruption, and more profound immune dysregulation in the spleen. Nevertheless, this interpretation remains speculative, as no direct correlational data between FDG uptake and splenic parasite load or histopathology exist to date. The underlying mechanisms warrant further dedicated study.

Among VL cases with evaluable PET/CT findings, approximately 6% showed no significant abnormalities, which may be attributable to the timing of the examination. For example, Antoine reported a case in which the initial PET/CT scan showed no abnormalities, but a follow-up scan approximately two months later demonstrated hypermetabolic activity in the bone marrow and spleen [13]. This suggests that negative PET/CT findings do not exclude VL, as early-stage disease may not yet manifest with positive findings. With disease progression, dynamic PET/CT monitoring may eventually reveal its typical characteristics.

PET/CT can also reveal the involvement of other rare sites in VL. Alghaythi et al. [15] reported a case presenting with increased uptake in the gastric antrum and colon; biopsy of a colonic lesion revealed cytomegalovirus colitis combined with VL. Vaitheeswaran et al. [16] reported a case that showed increased uptake in the intraspinal compartment (involving the spinal cord and meninges) and in subcutaneous soft tissue nodules. PCR analysis of the subcutaneous nodules and cerebrospinal fluid samples confirmed *Leishmania donovani*. Eichenberger et al. [48] reported a case with diffusely increased uptake in the colon, spleen, liver, lymph nodes, and basal lung segments, emphasizing that PET/CT can be used to evaluate the dissemination and severity of VL.

Case 1 was admitted and underwent a PET/CT scan, which revealed typical manifestations of RES involvement, characterized by increased FDG uptake in the spleen, bone marrow, cervical lymph nodes, and tonsils. Coupled with the epidemiological history—residence in a historically VL-endemic area adjacent to the currently VL-endemic Shanxi Province and the presence of dogs at home—these findings raised a strong suspicion of VL. Peripheral blood was promptly sent for mNGS testing, and a definitive diagnosis was confirmed within one week after the PET/CT scan. The time from symptom onset to diagnosis was approximately 3 weeks. Case 2 lived in a historically VL-endemic area, adjacent to the current VL-endemic region in Shanxi Province, and no dogs were kept at home. The patient was co-infected with EBV. The PET/CT scan showed only a few mildly hypermetabolic lymph nodes, with no typical signs of systemic RES involvement, leading to a diagnosis of HPS in the setting of EBV co-infection, after which chemotherapy was initiated. This misdiagnosis can be attributed to the clinician’s cognitive bias and failure to promptly recognize the epidemiological features of VL, along with the atypical clinical and PET/CT manifestations of VL and the complexity of the case. Bone marrow smears failed to reveal *Leishmania* amastigotes, and the diagnosis was ultimately confirmed by mNGS approximately 2.5 months after onset. Case 3 also resided in a historically VL-endemic area. However, the attending physician did not recognize this significant diagnostic clue and failed to inquire about the presence of dogs in the patient’s household. The patient’s PET/CT scan showed increased metabolism in the liver, spleen, bone marrow, and lymph nodes—typical manifestations of RES involvement. Nevertheless, the attending physician did not consider the possibility of VL. Due to elevated levels of certain rheumatic antibodies, the patient was misdiagnosed with a rheumatic disease and treated with corticosteroids for as long as three months. The diagnosis of VL was ultimately confirmed by peripheral blood mNGS approximately 4.5 months after symptom onset. This case highlights that clinicians should improve their recognition of VL PET/CT findings and attach great importance to the identification of VL epidemiological history. Furthermore, it illustrates that the typical PET/CT manifestations of VL involving the RES lack specificity and cannot be reliably differentiated from hematological disorders—particularly HPS—and connective tissue diseases, thereby easily leading to missed diagnosis and misdiagnosis. All three cases described above were ultimately diagnosed by peripheral blood mNGS, underscoring the significant value of molecular testing in the etiological diagnosis of VL. Bone marrow smear examination was performed in all four VL cases. In one case, the diagnosis was made by identifying *Leishmania* amastigotes on the smear; in two cases, no amastigotes were detected; and in the remaining case, although amastigotes were present, they were initially overlooked by the examiner. Only after the diagnosis was confirmed by mNGS did a retrospective review of the original bone marrow smear reveal the occasional presence of *Leishmania* amastigotes. According to the literature, among patients with documented results of initial bone marrow aspiration, amastigotes were not detected in 69% (44/64) of cases. This finding highlights the limitations of bone marrow smear microscopy for VL diagnosis, as its yield can be substantially influenced by factors such as smear quality, microscopist experience, sampling site, and parasite load. Therefore, a negative initial bone marrow examination should be interpreted with caution and does not rule out VL. None of the four VL patients underwent rK39 rapid diagnostic testing, suggesting that this method is not widely available in VL non-endemic areas.

In this study, Case 1 exemplified an ideal, efficient diagnostic pathway: the patient’s epidemiological history and PET/CT findings were relatively typical, leading clinicians to fully consider the possibility of VL, promptly submit samples for mNGS testing, and confirm the diagnosis. The time from symptom onset to diagnosis (approximately 3 weeks) was far shorter than that of Cases 2 and 3. This case demonstrates that when PET/CT findings are typical and correctly interpreted, they can provide crucial diagnostic clues. Similar to the successful experience of Case 1, in the case reported by Qiusha Guo et al. [12], the initial bone marrow smear showed only hemophagocytic phenomena without the detection of *Leishmania* amastigotes. The patient was initially diagnosed with HPS, but the treatment was ineffective, and splenectomy was recommended. However, PET/CT revealed increased FDG uptake in the spleen and bone marrow. Based on this finding, repeated bone marrow sampling identified a large number of *Leishmania* amastigotes, leading to a definitive diagnosis of VL-associated HPS. The patient was subsequently treated with sodium stibogluconate and achieved complete recovery, thereby avoiding splenectomy.

Case 2 and Case 3, on the other hand, represent different types of diagnostic delays, once again confirming the challenges of VL diagnosis: Case 2 was complicated by EBV infection, and its PET/CT findings lacked specificity (showing only increased uptake in several lymph nodes); Case 3’s PET/CT presented relatively typical manifestations of RES involvement, yet this key clue was long overlooked. A comparison of the diagnostic courses of the three cases in this group who underwent PET/CT clearly reveals the value and challenges of PET/CT in VL diagnosis: when imaging findings are typical and correctly interpreted (Case 1), they can provide crucial diagnostic clues; when findings are atypical (Case 2) or typical but unrecognized (Case 3), they may also lead to diagnostic delays. This suggests that clinical physicians should enhance their understanding of the PET/CT manifestations of VL. When PET/CT reveals typical involvement of the RES, the possibility of VL should be fully considered, and it is imperative to incorporate the epidemiological history and promptly conduct etiological examinations to shorten the diagnostic period. However, when PET/CT shows only lymphadenopathy without typical splenomegaly, increased uptake, or other manifestations, VL should still not be excluded.

In this review, 22 cases had a documented provisional diagnosis following PET/CT. The most commonly reported provisional diagnoses were malignant tumors and HPS. The nonspecific PET/CT appearance of VL overlaps substantially with that of other diseases, which may have contributed to these provisional diagnostic impressions. Focal FDG uptake in the spleen can be observed in metastatic malignancies, sarcoidosis, and sclerosing hemangioma-like nodular transformation. Diffuse FDG uptake in the spleen may be seen in leukemia, sarcoidosis, vasculitis, certain viral infections (e.g., EBV), protozoan infections (including leishmaniasis and malaria), and pharmacological interventions (e.g., granulocyte colony-stimulating factor, interferon α-2b) [33,64]. Diffuse increase in bone marrow FDG uptake is commonly observed following post-chemotherapy hematopoietic stimulation and may also be seen in leukemia and lymphoma [73]. The PET/CT findings of VL are easily confused with those of lymphoma. Multiple case reports have described patients who initially received a provisional diagnosis of “splenic lymphoma” due to negative *Leishmania* detection on bone marrow smears and PET/CT findings suggestive of splenic lymphoma, with VL ultimately confirmed only after splenectomy pathology [19,25,49]. Both VL and lymphoma can present with high metabolic activity in the spleen and bone marrow on FDG-PET/CT. However, VL typically exhibits more diffuse and homogeneous splenic uptake, milder and more localized lymph node involvement, and comparatively diffuse bone marrow uptake; furthermore, these abnormalities can completely resolve after treatment. In contrast, lymphoma tends to manifest as focal splenic lesions and is frequently accompanied by prominent lymph node involvement. Recent studies have demonstrated that connective tissue diseases, such as adult-onset Still’s disease (AOSD), frequently present with diffuse FDG uptake in the spleen and bone marrow, along with symmetric reactive lymph node hyperplasia in the cervical and axillary regions [78,79]. HPS may also manifest with splenomegaly and increased FDG uptake, together with increased bone marrow and lymph node uptake [80,81], making its differentiation from VL difficult.

This case series and a review of the prior literature indicate that when a patient with FUO presents with diffuse or multifocal increased FDG uptake in the spleen, and/or concomitant increased uptake in other RES organs, including the bone marrow, liver, and multiple regional lymph nodes, VL should be included in the differential diagnosis. Etiological evidence should be actively sought, particularly in patients with hepatosplenomegaly and/or pancytopenia, those originating from endemic regions, or those with a relevant travel history.

This study has the following limitations: First, the sample size is limited; although the analysis of the four cases that underwent PET/CT examination is informative, it does not permit statistical inference. Second, this is a retrospective case series, and indicators such as “time from onset to diagnosis” are influenced by the completeness of medical record documentation, potentially introducing recall bias. Third, the number of cases reported in the previous literature is relatively small, and the variability in FDG-PET/CT imaging due to differences in equipment and protocols, as well as the lack of standardized uptake descriptions, increases the heterogeneity of findings derived from the literature review. Fourth, the literature review conducted in this study is inherently subject to publication and selection bias. Published cases predominantly reflect those with unusual PET/CT findings, diagnostic challenges, or notable teaching value, whereas patients with unremarkable PET/CT manifestations or those with a straightforward clinical diagnosis of VL are substantially less likely to be reported. Fifth, in one included study, a minor numerical difference existed between the total of positive records listed in a detailed table (23) and the number of positive patients stated in the text (21). To maximize anatomical data, we retained all published positive records in the organ distribution and pattern analyses; for the immune-status subgroup, only cases with unequivocal linkage between immune status and scan result were included. Such unresolvable differences are an inherent limitation of literature-based reviews. Although a trivial bias could be introduced, its magnitude is minimal and does not affect the principal findings of this study. Consequently, the PET/CT imaging features summarized in this review may not fully represent the overall imaging spectrum of VL, and certain rare or striking signs may be overrepresented. Therefore, the viewpoints and diagnostic pathways proposed in this study require further validation through prospective, multicenter research. Nevertheless, given that VL is a relatively uncommon infectious disease, this study provides valuable clinical observations and insights, systematically elaborating on the utility of PET/CT in diagnosing VL, especially when the disease presents solely as FUO.

## 5. Conclusions

The typical PET/CT presentation of VL is characterized by increased RES FDG uptake, with or without enlargement of the liver, spleen, and lymph nodes, while involvement of other organs is not prominent. Integrating epidemiological history into the interpretation of PET/CT is critical for the timely and accurate diagnosis of VL. The typical PET/CT manifestations of VL can be easily confused with lymphoma, HPS, and other hematological disorders, as well as connective tissue diseases. Without correct interpretation in conjunction with epidemiological history, there is a risk of misdiagnosis or missed diagnosis. Therefore, before making a clinical diagnosis of the aforementioned disorders and initiating empirical treatments such as splenectomy, chemotherapy, or hormone therapy, VL should be considered and excluded. Of note, PET/CT can offer diagnostic clues, but confirmation generally relies on other modalities. Additionally, PET/CT may be useful in screening for comorbidities, guiding targeted biopsy, assessing systemic disease distribution, and monitoring treatment response in VL, but these potential uses remain to be further studied.

## Figures and Tables

**Table 1:** Comparison of epidemiological history and general clinical characteristics in four cases of VL.

Case	From Historical VL-Endemic Areas	Dog Ownership	Symptoms and Signs at Admission	Conventional Imaging Findings	Purpose of PET/CT Scan
1	Yes	Yes	Fever (2 weeks, peak 39.5 °C) with chills/rigors; physical examination: normal.	Hepatomegaly and splenomegaly; visible cervical, bilateral axillary, and inguinal lymph nodes (no atypia).	Etiologic Screening for FUO
2	Yes	No	Fever (6 days, intermittent, peak 39.8 °C); physical examination: pallor, hepatomegaly, splenomegaly (4 cm below costal margin).	Hepatomegaly, splenomegaly, and multiple lymph nodes in the right supraclavicular fossa, both axillae, and both inguinal regions.	Etiologic Screening for FUO
3	Yes	Not Recorded	Fever (2 weeks, intermittent, peak 39.0 °C) with chills/rigors, fatigue, night sweats; physical examination: normal.	Mild hepatosplenomegaly; visible bilateral inguinal lymph nodes; visible bilateral cervical lymph nodes.	Etiologic Screening for FUO
4	Yes	Not Recorded	No fever; physical examination: bilateral small axillary and inguinal lymphadenopathy	Splenomegaly	Confirmed VL; evaluation for lymphoproliferative disorders (elevated serum immunoglobulins and a positive serum immunofixation electrophoresis).

Note: VL, visceral leishmaniasis; FUO, fever of unknown origin.

**Table 2:** Comparison of PET/CT manifestations, diagnostic and therapeutic decision-making process in four cases of VL.

Case	Detailed PET/CT Findings	Clinical Interpretation and Decision-Making	Time from PET/CT to mNGS Decision	Time from Onset to Diagnosis
1	1. Splenomegaly with mildly increased metabolic activity (SUVmax: 4.6); 2. Diffuse, heterogeneous mildly increased metabolic activity in the bone marrow region (SUVmax range: 2.0–5.7); 3. Multiple lymph nodes in both sides of the neck with mildly to highly increased metabolic activity, some slightly enlarged (SUVmax range: 2.3–12.8); 4. Bilateral prominent tonsils, more pronounced on the right side (SUVmax: 14.9).	Strong suspicion of VL; immediate decision for mNGS testing.	Brief (less than one week)	approximately 3 weeks
2	1. Splenomegaly was noted without accompanying abnormal hypermetabolism; 2. Scattered small lymph nodes were noted in both axillae and both inguinal regions, some with mild hypermetabolism (SUVmax of approximately 1.5), considered benign; 3. Multiple subcutaneous effusions were noted within the imaged area, accompanied by mild hypermetabolism, likely representing edema.	Only HPS considered and chemotherapy administered; VL not considered.	Prolonged (approximately 1.5 months)	approximately 2.5 months
3	1. Mild splenomegaly with diffuse moderately increased metabolic activity (SUVmax range: approx. 4.4–8.0); 2. Diffuse mildly increased metabolic activity in the bone marrow (SUVmax range: 1.1–3.7); 3. Slightly prominent liver with mildly increased metabolic activity (SUVmax range: approx. 2.5–4.6); 4. Several lymph nodes with mildly increased metabolic activity (SUVmax range: approx. 2.0–11.2) noted adjacent to the common hepatic artery, in the portacaval space, along the right external iliac vessels, and in the right inguinal region; some of these nodes show mild cortical thickening.	Misdiagnosed as HPS and autoimmune disease; long-term corticosteroid therapy administered; VL not considered.	Extremely prolonged (approximately three months)	approximately 4.5 months
4	1. Multiple lymph nodes in the bilateral neck, bilateral axillae, mediastinum, bilateral hilar regions, right cardiophrenic angle, retroperitoneal para-aortic region, mesenteric space, and bilateral inguinal regions; some of them are enlarged, with abnormally increased metabolic activity (SUVmax range: 1.6–31.4); 2. Splenomegaly with mildly abnormal increased metabolic activity (SUVmax: 3.2); 3. Mildly increased metabolic activity in the bone marrow of the bilateral ribs, scapulae, vertebrae, and pelvic bones (SUVmax range: 2.4–4.5).	PET/CT used after VL diagnosis to evaluate for concomitant hematological diseases and to successfully guide biopsy.	Not applicable	Not applicable

Note: Time from PET/CT to mNGS Decision: interval from the date the clinical team received the PET/CT report to the date the decision was made to send peripheral blood for mNGS testing. Time from Onset to Diagnosis: interval from the first documented onset of VL-attributable symptoms to the date VL was confirmed (by positive mNGS and/or established diagnostic criteria). All intervals are expressed in the most appropriate unit (weeks or months) for each case. VL, visceral leishmaniasis; SUVmax, maximum standardized uptake value; mNGS, metagenomic next-generation sequencing; HPS, hemophagocytic syndrome.

**Table 3:** Classification of reported PET/CT findings in VL cases according to explicit FDG uptake description.

Category	Definition	Number of Cases
Explicitly Positive	Abnormal FDG uptake explicitly reported.	83
Explicitly Negative	Absence of abnormal uptake explicitly stated.	5
Non-evaluable	FDG uptake not reported, or suspicion based solely on CT morphology without metabolic descriptor, FDG uptake confined to VL-incompatible sites.	11
Total number		99

**Table 4:** Combination Patterns of Organs with Explicitly Reported Abnormal FDG Uptake on PET/CT in VL Cases.

Combination Patterns	Number of Cases
Spleen and bone marrow	23
Spleen only	20
Spleen, bone marrow, and lymph nodes	8
Spleen and lymph nodes	6
Spleen, liver, and bone marrow	6
Lymph nodes only	5
Spleen, liver, and lymph nodes	2
Spleen, liver, lymph nodes, and bone marrow	2
Spleen, bone marrow, lung and/or pleura	1
Bone marrow and lymph nodes	1
Spleen, lymph nodes, lung and/or pleura, colon	1
Spleen, liver, lymph nodes, lung and/or pleura, colon	1
Liver only	1
Spleen, liver, bone marrow, lymph nodes, and tonsils	1
Spleen, liver, bone marrow, lung and/or pleura	1
Spleen, liver, lymph nodes, and conjunctiva	1
Lymph nodes, subcutaneous soft tissue nodules, and intraspinal canal	1
Colon and stomach	1
Bone marrow only	1
PET/CT demonstrated no abnormal uptake	5
Total number	88

Note: PET/CT: positron emission tomography/computed tomography. This section was performed excluding the 11 Non-evaluable cases. Pattern classification was based on organs with explicitly reported abnormal FDG uptake. Only organs clearly described as positive are listed; organs with unreported or unclear information were not considered positive. Because most source reports do not systematically document uninvolved organs, the absence of an organ in this table does not indicate confirmed negative status. Detailed original descriptions and SUVmax values are provided in Supplementary Table S1. In the study by Zanoni et al. [44], the detailed organ involvement table lists 23 PET/CT-positive cases, whereas the text explicitly states that 21 of 23 patients were positive (the remaining 2 being immunocompromised and PET/CT-negative). As the source of the discrepancy cannot be identified, we retained all 23 positive records to avoid discarding potentially valid anatomical data.

## Data Availability

The original contributions presented in this study are included in the article/Supplementary Material. Further inquiries can be directed to the corresponding authors.

## Supplementary Materials

The following supporting information can be downloaded at: https://www.mdpi.com/article/10.3390/diagnostics16203402/s1, Table S1: Summary of detailed information for all cases identified through the literature review is located in the Supplementary Materials (Table S1‑A. Summary of detailed information for all cases identified through the literature review—cases classified as “Explicitly Positive” on FDG‑PET/CT, Table S1‑B. Summary of detailed information for all cases identified through the literature review — cases classified as “Explicitly Negative” on FDG‑PET/CT, Table S1‑C. Summary of detailed information for all cases identified through the literature review — cases classified as “Non‑evaluable” on FDG‑PET/CT). References [7,8,9,10,11,12,13,14,15,16,17,18,19,20,21,22,23,24,25,26,27,28,29,30,31,32,33,34,35,36,37,38,39,40,41,42,43,44,45,46,47,48,49,50,51,52,53,54,55,56,57,58,59,60,61,62,63,64,65,66,67,68,69,70,71,72,73,74,75,76,77] are cited in the supplementary materials.

## Abbreviations

VL	visceral leishmaniasis
mNGS	metagenomic next-generation sequencing
PET/CT	^18^F-fluorodeoxyglucose positron emission tomography/computed tomography
FUO	fever of unknown origin
RES	reticuloendothelial system
HPS	hemophagocytic syndrome
FDG	^18^F-fluorodeoxyglucose
SUV	standardized uptake value
SUVmax	maximum standardized uptake value
PCR	polymerase chain reaction
EBV	Epstein–Barr virus

## References

[1] Bamorovat M., Sharifi I., Khosravi A., Aflatoonian M.R., Agha Kuchak Afshari S., Salarkia E., Sharifi F., Aflatoonian B., Gharachorloo F., Khamesipour A. (2024). Global Dilemma and Needs Assessment Toward Achieving Sustainable Development Goals in Controlling Leishmaniasis. J. Epidemiol. Glob. Health.

[2] Lun Z.-R., Wu M.-S., Chen Y.-F., Wang J.-Y., Zhou X.-N., Liao L.-F., Chen J.-P., Chow L.M.C., Chang K.P. (2015). Visceral Leishmaniasis in China: An Endemic Disease under Control. Clin. Microbiol. Rev..

[3] Li Y., Luo Z., Hao Y., Zhang Y., Yang L., Li Z., Zhou Z., Li S. (2022). Epidemiological features and spatial-temporal clustering of visceral leishmaniasis in mainland China from 2019 to 2021. Front. Microbiol..

[4] World Health Organization (2010). Control of the Leishmaniasis: Report of a Meeting of the WHO Expert Committee on the Control of Leishmaniases, Geneva, 22–26 March 2010.

[5] Sarfraz A., Sarfraz Z., Liaqat M., Khan M.H., Abdul Jabbar H.A., Abdullah M. (2024). Leishmaniasis—Still a diagnostic challenge: An individual participant data systematic review. Trop. Dr..

[6] China General Office of the National Health Commission of the People’s Republic of China, Department of General Affairs, National Administration of Traditional Chinese Medicine of the People’s Republic of China (2023). Diagnosis and Treatment Protocol for Kala-azar.

[7] Ceylan S., Mert A. (2025). A Case of Kala-azar Diagnosed via PET/CT and Confirmed by Splenectomy. Med. Sci. Discov..

[8] Arzel M., Bellini C., Milowich D., Nabergoj M. (2025). Visceral Leishmaniasis Mimicking Aggressive Lymphoma. Cureus.

[9] Gestels N., Bottieau E., De Vooght A., van Ierssel S., Libbrecht S., Driessen A., Francque S., Vanwolleghem T., Kwanten W.J. (2025). An unusual case of acute cholestatic hepatitis: Visceral leishmaniasis with secondary haemophagocytic lymphohistiocytosis. Acta Gastroenterol. Belg..

[10] Ibad I.K., Vankayala A., Manwatkar A., Mathew J. (2025). Visceral leishmaniasis mimicking inflammatory myositis that led to haemophagocytic lymphohistiocytosis. BMJ Case Rep..

[11] Wang H., Li J., Huang W. (2025). Case Report: Fever of unknown origin with hemophagocytic lymphohistiocytosis and intestinal hemorrhage—A successfully treated case of severe visceral leishmaniasis. Front. Med..

[12] Guo Q., Liu X. (2025). FDG PET/CT enables splenectomy avoidance in visceral leishmaniasis-associated hemophagocytic lymphohistiocytosis: A case report. Asian J. Surg..

[13] Decaestecker A., Daniel A., Malbranque Z., Wayolle N., Ydee A. (2025). Crescentic glomerulonephritis associated with myelodysplatic syndrome and visceral leishmaniasis. J. Nephrol..

[14] Battino N., Pisano A. (2025). Visceral leishmaniasis and lymphoma: A rare and dangerous couple. Ital. J. Med..

[15] Alghaythi S., Almuhaidb A., Al-Bawardy B. (2025). Double Trouble: Leishmaniasis and Cytomegalovirus Colitis in an Immunocompromised Host. ACG Case Rep. J..

[16] Kumar Vaitheeswaran K., Gupta B.K., Krishnan G R., Soneja M., Vikram N.K., Baitha U., Singh A., Wig N., Azam M., Singh R. (2024). Neuro-leishmaniasis with cauda equina syndrome and cranial nerve palsy: A rare manifestation of recurrent atypical visceral leishmaniasis. BMC Infect. Dis..

[17] Chang P., Al-Shyoukh A., Grewal H., Burke V. (2024). An Uncommon Cause of a Common Finding: Mediastinal Lymphadenopathy Secondary to Leishmaniasis. Am. J. Respir. Crit. Care Med..

[18] Banegas D.E., Moioli A., Santoni E., Tagliavini E., Quaglia F.M., Bernardelli A., Visco C. (2024). Visceral Leishmaniasis Following A+AVD Treatment in a Patient with Classical Hodgkin’s Lymphoma: A Case Report and Review of the Literature. J. Clin. Med..

[19] Yang M., Xiang B., Wei T., Chi Y.e., Li J. (2024). Visceral leishmaniasis complicated by myelofibrosis: A case report. Chin. J. Parasitol. Parasit. Dis..

[20] Ea V., Papa B., Goldberg R. (2024). A case of visceral leishmaniasis masquerading as autoimmune hepatitis. Med. J. Aust..

[21] Jancar N., Sousa Goncalves F., Duro J., Pinto I., Oliveira T., Aguiar P. (2023). Kala-Azar: A Case Report. Cureus.

[22] Tolaj I., Mehmeti M., Gashi H., Berisha F., Gashi V., Fejza H., Shala N. (2023). Visceral leishmaniasis in Kosovo: A case of misdiagnosis and diagnostic challenges. IDCases.

[23] Wang L., Hu M., Wu X., Ma L., Yang H. (2023). Case Report: Diagnosis and Treatment of Two Clinical Cases of Visceral Leishmaniasis-Related Hemophagocytic Lymphohistiocytosis. Am. J. Trop. Med. Hyg..

[24] Gulati S., Dessai R., Patnaik N.K., Chawla K. (2023). Visceral leishmaniasis escaping the diagnosis and withstanding treatment in a case of recurrent pyrexia. Int. J. Res. Med. Sci..

[25] Feng H., Dai W. (2023). Case Report: FDG-PET/CT findings in co-infection of visceral leishmaniasis and chronic hepatitis B. Front. Cell. Infect. Microbiol..

[26] Liu Y., Guo Z., Sun L., Zhang D. (2023). Clinical and epidemiological investigation and analysis of a new visceral leishmaniasis case in Xin’an County, Henan Province. Dis. Surveill..

[27] Huang S., Zhao J., Li H., Chen A., Zhang X., Tang R., Yang R. (2023). Adult kala-azar complicated by multiple organ dysfunction syndrome: Report of 3 cases. Jiangsu Med. J..

[28] Porto V.B.G., Carvalho L.B., Buzo B.F., Litvoc M.N., Santos A.C.S., Rocci R.A., Soares S.R.C., Zampieri R.A., Duarte M.I.S., Lindoso J.A.L. (2022). Visceral leishmaniasis caused by Leishmania (Leishmania) amazonensis associated with Hodgkin’s lymphoma. Rev. Inst. Med. Trop. São Paulo.

[29] Solimando A.G., Coniglio G., Desantis V., Lauletta G., Bavaro D.F., Diella L., Cirulli A., Iodice G., Santoro P., Cicco S. (2022). A Challenging Case of Visceral Leishmaniasis. Reports.

[30] Song Q., Sheng S., Wang Y., Shao J., Li T. (2022). Investigation and analysis of a kala-azar case in Wuxi City. Jiangsu J. Prev. Med..

[31] Zaghi I., Ielasi L., Stagni B., Cascavilla A., Ferri S., Ambrosini V., Riefolo M., Varani S. (2022). A case of Leishmania infection with focal splanchnic involvement without systemic symptoms: A potential anti-inflammatory role for vitamin D. Acta Clin. Belg..

[32] Dunzinger A., Datinger C., Loidl A., Walcherberger B., Lengauer R.A., Mehraban N., Pichler R. (2022). Visceral Hepatic Leishmaniasis in a Melanoma Patient in FDG-PET. Curr. Med. Imaging Rev..

[33] Pinnegar H.P., Sánchez-Montalvá A., Barios Profitos M., Bosch-Nicolau P., Salvador F., Molina I. (2021). Utility of Fluorine-18 Fluorodeoxyglucose Positron Emission Tomography/Computed Tomography in Patients with Visceral Leishmaniasis: Case Report and Literature Review. Am. J. Trop. Med. Hyg..

[34] Shi Q., Huang M., Li X., Zheng X., Wang F., Zou Y., Wang L., Jia J. (2021). Clinical and laboratory characteristics of hemophagocytic lymphohistiocytosis induced by Leishmania infantum infection. PLoS Neglected Trop. Dis..

[35] Zou L., Chen G., Zhou Y., Ye W., Wen Y., Chen L., Li X. (2021). Continuous hypergammaglobulinemia and proteinuria after the recovery of the visceral Leishmaniasis: A case report. BMC Infect. Dis..

[36] Patoulias D., Papachristou S., Gouridou E., Stamou D., Chissan S., Spyridon B. (2021). A Patient with Rheumatoid Arthritis under Methotrexate and Etanercept Treatment Presenting with Fever and Pancytopenia: An Unexpected Guest. Mediterr. J. Rheumatol..

[37] Liu H., Fan H., Huang X., Jiao Y. (2021). The clinical characteristics and outcomes of patients with fever of unknown origin caused by parasitic infection. Medicine.

[38] Meng G., Wang J., Wu L., Wang Z. (2020). Visceral leishmaniasis complicated by hemophagocytic lymphohistiocytosis: A case report. Chin. J. Infect. Chemother..

[39] Zhang G., Zhong J., Wang T., Zhong L. (2020). A case of visceral leishmaniasis found by left oblique hernia: A case report. Exp. Ther. Med..

[40] Sayin Kutlu S., Kutlu M., Herek D., Dirim Erdogan D., Ozdemir K., Sen Turk N., Yaylali O., Hacıoglu S., Turgut H. (2019). Multiple Nodular Lesions in Spleen and Liver in Visceral Leishmaniasis. Infect. Dis. Clin. Microbiol..

[41] Martinez de Narvajas I., Diaz A., Bassegoda O., Carpio A., Fuster C., Valls M.E., Alvarez-Martinez M.J., Garcia-Vidal C., Soriano A., Martinez J.A. (2019). Acute liver failure due to visceral leishmaniasis in Barcelona: A case report. BMC Infect. Dis..

[42] Machelart I., Lapoirie J., Viallard J.F., Mirabel M., Lazaro E., Greib C., Rivière E. (2019). Visceral leishmaniasis with hemophagocytic lymphohistiocytosis in an immunocompetent adult. Médecine Mal. Infect..

[43] Rinaldi F., Giachè S., Spinicci M., Corsi P., Ambu S., Gianfaldoni G., Rigacci L., Arena U., Bartoloni A., Zammarchi L. (2019). Focal spleen lesions in visceral leishmaniasis, a neglected manifestation of a neglected disease: Report of three cases and systematic review of literature. Infection.

[44] Zanoni L., Varani S., Attard L., Morigi J.J., Vanino E., Ortalli M., Fonti C., Viale P., Re M.C., Fanti S. (2019). 18F-FDG PET/CT in visceral leishmaniasis: Uptake patterns in the context of a multiannual outbreak in Northern Italy. Ann. Nucl. Med..

[45] Schwetz V., Trummer C., Friedl C., Beham-Schmid C., Kulnik R., Wolfler A., Horvath K., Wunsch S., Prattes J., Zollner-Schwetz I. (2018). Visceral leishmaniasis in a patient with diabetes mellitus type 2 and discrete bicytopenia. Clin. Case Rep..

[46] Delfos N.M., den Hartog W.C.E., Anten S. (2018). Koorts uit onverwachte hoek. Tijdschr. Infect..

[47] Tatarelli P., Fornaro G., Del Bono V., Nicolini L.A., Raiola A.M., Gualandi F., Varaldo R., Di Muccio T., Gramiccia M., Gradoni L. (2018). Visceral leishmaniasis in hematopoietic cell transplantation: Case report and review of the literature. J. Infect. Chemother..

[48] Eichenberger A., Buechi A.E., Neumayr A., Hatz C., Rauch A., Huguenot M., Diamantis-Karamitopoulou E., Staehelin C. (2017). A severe case of visceral leishmaniasis and liposomal amphotericin B treatment failure in an immunosuppressed patient 15 years after exposure. BMC Infect. Dis..

[49] Feifei L., Rugui L., Fang L. (2017). Clinical analysis of a couple with kala-azar complicated by splenic infarction. Chin. J. Clin. Infect. Dis..

[50] Darcis G., Van der Auwera G., Giot J.-B., Hayette M.-P., Tassin F., Arrese Estrada J., Cnops L., Moutschen M., de Leval L., Leonard P. (2017). Recurrence of visceral and muco-cutaneous leishmaniasis in a patient under immunosuppressive therapy. BMC Infect. Dis..

[51] Wu Y., Meng J., Zhang X., Chen J., Luan C., Tang G., He Z. (2017). Hemophagocytic syndrome induced by kala-azar: A case report and literature review. J. Clin. Hematol..

[52] Prajapati R., Kumar A., Sharma P., Singla V., Bansal N., Dhawan S., Arora A. (2016). A Rare Presentation of Leishmaniasis. J. Clin. Exp. Hepatol..

[53] Chianura L., De Ferrari M.E., Popescu C.E., Cantoni S., Colussi G., Rossetti C., Puoti M. (2014). The role of F-18 FDG PET in the diagnosis of visceral leishmaniasis: Two case reports. J. Case Rep..

[54] Comont T., Martin-Blondel G., Laurent C., Berry A., Marchou B. (2014). Severe haemophagocytic lymphohistiocytosis triggered by a visceral leishmaniasis in a patient with a Rosai-Dorfman disease. Ann. Hematol..

[55] Fuertes J., Garcia-Bennett J.R., Iftimie S., Danús M., Abreu J.A. (2014). Focal splenic FDG uptake in a patient with Kala-Azar (visceral leishmaniasis). Clin. Nucl. Med..

[56] Gallina V., Binazzi R., Golemi A., Farsad M., Weiss G., Wiedermann C.J. (2014). Imported visceral leishmaniasis—Unexpected bone marrow diagnosis in a patient with fever, pancytopenia, and splenomegaly. Am. J. Blood Res..

[57] Guard D., Nigam S. (2014). 23. Case study: Visceral leishmaniasis—A rare cause of hepatosplenomegaly in australia. Pathology.

[58] Mao G., Yang G., Cheng Y., Zee C.S., Huang W., Ni W., Meng G., Chen Z. (2014). Multiple Nodular lesions In Spleen Associated With Visceral Leishmaniasis. Medicine.

[59] van Raalte D.H., Wesselius H.M., de Klerk G. (2014). Unexpected diagnosis of visceral leishmaniasis in a patient resenting with an infected ICD lead. Neth. J. Med..

[60] Pan Y., Kang H., Liu G., Liu Y., Li M., Wang C. (2014). Report of 3 misdiagnosed cases of kala-azar. Acad. J. Chin. PLA Med. Sch..

[61] Molinaro F., Varriale A., De Matthaeis A., De Cata A., Scalzulli P. (2014). Leishmaniasis: A misunderstood case of FUO. Ital. J. Med..

[62] Murri M., Fraticelli P., Goteri G., Mattioli M., Kafyeke A., Martino G.P., Scortechini I., Leoni P., Gabrielli A. (2014). Visceral leishmaniasis in a patient with Coombs negative haemolytic anaemia and polycythemia vera:a case report. Ital. J. Med..

[63] Morigi J.J., Attard L., Ambrosini V., Salvadori C., Nanni C., Tabacchi E., Ghedini P., Ceci F., Cambioli S., Viale P. (2014). Diagnosing visceral leishmaniasis through PET/CT: Possible distribution patterns. Eur. J. Nucl. Med. Mol. Imaging.

[64] Gibson G.M., Arnold C., Kumar A.S.R. (2014). 18F-FDG Uptake in Multiple Splenic Foci on PET/CT An Unusual Case of Visceral Leishmaniasis. Clin. Nucl. Med..

[65] Evers G., Pohlen M., Berdel W.E., Thoennissen N.H., Titze U., Köhler G., Weckesser M., Anthoni C., Mesters R.M. (2013). Visceral leishmaniasis clinically mimicking lymphoma. Ann. Hematol..

[66] Berry C.E., Tsai J., Tierney A., Pickles R. (2013). Visceral leishmaniasis in a patient taking adalimumab for rheumatoid arthritis. Med. J. Aust..

[67] Wu L., Wang Y., Cui H., Wang Z. (2013). Hemophagocytic lymphohistiocytosis induced by Leishmania infection. J. Intern. Med. Concepts Pract..

[68] Kyrtatos P.G., Debard A., Martin-Blondel G., Alvarez M., Delobel P., Marchou B., Massip P., Wagner T. (2013). FDG-PET/CT findings during immune reconstitution in an HIV-1 patient infected with visceral leishmaniasis. Infection.

[69] Ravaglia C., Gurioli C., Casoni G.L., Asioli S., Poletti V. (2013). Fever, splenomegaly and lymphopenia in sarcoidosis. Thorax.

[70] Martin C., Castaigne C., Tondeur M., Flamen P., De Wit S.J.H.m. (2013). Role and interpretation of fluorodeoxyglucose-positron emission tomography/computed tomography in HIV-infected patients with fever of unknown origin: A prospective study. HIV Med..

[71] Fan F., Su Y., Wang Y., Liu Y., Yi H., Fu L., Qiu L. (2012). Kala-azar with hemophagocytic syndrome as the initial manifestation: A case report. J. Third Mil. Med. Univ..

[72] Vase M.O., Hellberg Y.K., Larsen C.S., Petersen E., Schaumburg H., Bendix K., Ravel C., Bastien P., Christensen M., D’Amore F. (2012). Development of splenic marginal zone lymphoma in a HIV-negative patient with visceral leishmaniasis. Acta Haematol..

[73] Yapar A.F., Reyhan M., Kocer N.E., Aydin M., Nursal G.N. (2011). Diffuse Splenic F-18 FDG Uptake in Visceral Leishmaniasis. Clin. Nucl. Med..

[74] Bielory B.P., Lari H.B., Mirani N., Kapila R., Fitzhugh V.A., Turbin R.E. (2011). Conjunctival Squamous Cell Carcinoma Harboring Leishmania Amastigotes in a Human Immunodeficiency Virus–Positive Patient. Arch. Ophthalmol..

[75] Casadevall M., Mambré L., Cornet M., Caillat-Vigneron N., Le Jeunne C., Aslangul E. (2010). Contribution of 18-FDG positron emission tomography for the diagnosis of visceral leishmaniasis. Médecine Mal. Infect..

[76] Lupi A., Todeschini G., Zanco P. (2006). Diffuse metabolic activation of reticuloendothelium on F-18 FDG PET imaging in a case of visceral leishmania. Clin. Nucl. Med..

[77] Myles O., Wortmann G., Barthel R., Ockenhouse C., Centers for Disease Control and Prevention (2004). Two Cases of Visceral Leishmaniasis in U.S. Military Personnel—Afghanistan, 2002–2004. Morb. Mortal. Wkly. Rep..

[78] Chen Z., Li Y., Wang Q., Weng S., Zhou Y., Zhu J. (2021). Fluorine-18 labeled fluorodeoxyglucose positron emission tomography/computed tomography used in diagnosing connective tissue diseases in fever of unknown origin/inflammatory of unknown origin patients. Clin. Rheumatol..

[79] Zhou X., Li Y., Wang Q. (2020). FDG PET/CT used in identifying adult-onset Still’s disease in connective tissue diseases. Clin. Rheumatol..

[80] Aggarwal P., Tyagi L., Singh H., Kumar R., Singh H., Prakash G., Jindal A.K., Sachdeva M.S., Suri D., Mittal B.R. (2025). Utility of 18F FDG PET/CT in Hemophagocytic Lymphohistiocytosis. Indian J. Nucl. Med..

[81] Yang X., Lu X., Feng L., Wang W., Kan Y., Zhang S., Li X., Yang J. (2024). Enhancing diagnostic precision in EBV-related HLH: A multifaceted approach using 18F-FDG PET/CT and nomogram integration. Cancer Imaging.

