# Supplementary material for: Application Value and Cognitive Challenges of PET/CT in the Diagnosis and Treatment of Visceral Leishmaniasis: A Retrospective Single-Center Case Series and Literature Review
Source: Diagnostics (Basel). 2026 Oct 19;16(20):3402. doi: 10.3390/diagnostics16203402 (PMC13703260; doi:10.3390/diagnostics16203402)
Supplement: Supplementary file 1 [file diagnostics-16-03402-s001.zip › diagnostics-4554291-supplementary.pdf]

**Table S1-A.** Summary of detailed information for all cases identified through the literature review — cases classified as "Explicitly Positive" on FDG-PET/CT

| Au-thor(s), Year, Region of consultation | Age (years), Sex | Underlying disease | Im-muno-sup-pres-sion | Indi-ca-tion for PET/CT | In-i-tial BM smear/bi-opsy: Leishmania amastigotes de-tected | General Imaging and Physical Examination Findings for Hepatomegaly, Splenomegaly, and Lymphadenopathy | PET/CT findings | Diagnosis after PET/CT | Time from PET/CT to VL confirmation | Process and methods for VL confirmation after PET/CT |
|---|---|---|---|---|---|---|---|---|---|---|
| Sule Ceylan, 2025. Turkey [1] | 48, M | NR | No | FUO etiol-ogy in-ves-tiga-tion | No | Sple-no-meg-aly | 1. Splenomegaly with markedly in-creased, diffusely heterogeneous splenic FDG uptake and an elevated spleen-to-liver SUVmax ratio.<br>2. Heterogeneous FDG uptake in the bone marrow. | Primary splenic lymphoma | NR | Splenectomy was performed. Histopathological examination of the spleen tissue revealed intracytoplasmic amastigotes. Postoperative ELISA confirmed the presence of *Leishmania* IgG antibodies. |
| Marie Arzel, 2025. Switzerland [2] | 48, M | Asthma | No | FUO etiol-ogy in-ves-tiga-tion | No | Hepatosple-no-meg-aly | 1. Hypermetabolic splenomegaly (SUVmax 14.7).<br>2. Homogeneous hypermetabolism in the liver and diffuse hypermetabolism in the bone marrow<br>3. Subcentimeter hypermetabolic lymph nodes in the right axilla; mildly hypermetabolic lymph nodes i | Lym-phoproliferative disor-ders | NR | Bone marrow biopsy, liver biopsy, and flow cytometric analysis of peripheral blood and bone marrow fluid revealed no evidence of lymphoma. Given the patient's travel history to an endemic area, VL was suspected. |

| | | | | | | | | | | |
|---|---|---|---|---|---|---|---|---|---|---|
| | | | | | | | n the neck and right subpectoral region. 4. Nonspecific hypermetabolism in the bilateral tonsils. | | | PCR tests for *Leishmania* on blood and bone marrow specimens were positive, and re-examination of the bone marrow smear revealed amastigotes. |
| N. Gestels, 2025. Belgium[3] | 64, M | Gastroesophageal reflux disease | No | NR | No | Hepatosplenomegaly | 1. Splenomegaly with heterogeneous, markedly increased FDG uptake. 2. Several FDG-positive lymph nodes in the retroperitoneal space. | HPS of unknown etiology | NR | The PCR test result was positive for *Leishmania*, and the species was subsequently identified as *Leishmania infantum*. |
| Qiusha Guo, 2025. China [4] | 44, F | None | No | NR | No | Splenomegaly | 1. The enlarged spleen exhibits diffusely increased FDG uptake; the SUVmax of the most active splenic areas is 3.0, slightly higher than the liver SUVmax (2.5). 2. The bone marrow demonstrates diffusely increased FDG uptake, with an SUVmax of 3.4. | NR | NR | Based on the PET/CT conclusions, repeated bone marrow sampling revealed numerous *Leishmania* parasites, thereby avoiding splenectomy. |
| Antoine Decaestecker, 2025. France [5] | 53, M | Left nephrectomy for clear cell renal cell carcinoma (10 years prior); myelodysplastic syndrome with corticosteroid-sensitive autoinflammatory manifestations (relapsing polychondritis, scleritis, and pericarditis), VEXAS-like (UBA | NR | NR | No | NR | While the initial examination revealed no hypermetabolic foci, the second demonstrated hypermetabolic activity in the bone marrow and spleen. | NR | NR | The second bone marrow examination revealed numerous *Leishmania* parasites, and *Leishmania* PCR of blood was strongly positive. Re-examination of the initial bone marrow smear detected a small number of *Leishmania* parasites. |

| | | | | | | | | | | |
|---|---|---|---|---|---|---|---|---|---|---|
| | | 1 mutation-negative), under observation | | | | | | | | |
| Nicola Battino, 2025. Italy[6] | 54, M | None | No | NR | Yes | Hepatosplenomegaly | 1. Increased glucose metabolism in the liver and spleen.<br>2. Widespread hypermetabolic activity in the medullary cavity.<br>3. Radiotracer accumulation in the area of consolidation in the right lower lung. | NR | NR | Bone marrow biopsy simultaneously detected *Leishmania* parasites and intravascular large B-cell lymphoma. |
| Saleh Alghaythi, 2025. Saudi Arabia [7] | 73, M | renal transplantation, type 2 diabetes, hypertension, ischemic heart disease, primary benign prostatic hyperplasia | Yes (corticosteroids, immunosuppressants) | Investigation of constitutional symptoms | NR | No hepatosplenomegaly; mesenteric lymphadenopathy. | 1. Increased metabolic activity in the gastric antrum.<br>2. A few enlarged mesenteric lymph nodes, all less than 1 cm in size.<br>3. One lesion with increased FDG uptake in the hepatic flexure of the colon. | NR | NR | Biopsy of a transverse colon polyp revealed cytomegalovirus colitis concurrent with leishmaniasis. |
| Karthick Kumar Vaitheeswaran, 2024. India [8] | 38, M | None | No | NR | Yes | Generalized lymphadenopathy; no hepatosplenomegaly. | 1. Multiple FDG-avid lymph nodes were observed in the bilateral cervical, bilateral axillary, mediastinal, retroperitoneal, mesenteric, bilateral external iliac, and bilateral inguinal regions.<br>2. Several well-defined subcutaneous soft tissue nodules with increased FDG uptake were noted on the left chest wall, corresponding to the left 6th intercostal space.<br>3. Increased FDG uptake was observed within the spinal canal (involving the spinal cord and meninges), extending from the 10th thora | NR | NR | Bone marrow and lymph node biopsies revealed Leishman–Donovan bodies; rK39 serology of cerebrospinal fluid was positive; lymph node biopsy samples yielded *Leishmania* on culture; and PCR of subcutaneous nodules, lymph nodes, and cerebrospinal fluid detected *Leishmania donovani*. |

| | | | | | | | | | | |
|---|---|---|---|---|---|---|---|---|---|---|
| | | | | | | | cic vertebra to the 1st sacral verte bra. After treatment, follow-up PE T/CT showed complete regression of the lesion. | | | |
| P. Cha ng, 2024. Americ a[9] | 47, M | HIV infec-tion, diffuse large B-cell lymphoma | Yes (HIV infec-tion, chem-other-apy for malig-nancy) | NR | NR | Medi-astinal lym-phad-e-nopa-thy; hepato sple-no-meg-aly; mes-en-teric lym-phad-e-nopa-thy. | The mediastinal, hilar, and mesen-teric lymph nodes exhibit increased metabolic activity. | NR | NR | Lymph node biopsy revealed no tumor, and cytologic ex-amination of bronchoalveolar lavage fluid revealed *Leish-mania donovani* within histi-ocytes. |
| Daniela Estefa nia Ba negas, 2024. Italy[10] | 43, M | Nodular sclerosis stage IV classical Hodgkin lymphoma | Yes (chem other-apy for malig-nancy) | Asse ssme nt fo r Ly mph oma Rela pse Statu s | NR | Sple-no-meg-aly | Splenomegaly with increased $^{18}$F-FDG uptake (SUVmax 6.7). | NR | NR | *Leishmania* PCR of periph-eral blood was positive. |
| Mei Ya ng, 2024. China [11] | 15, M | NR | NR | NR | No | Hepat osplen omega ly wit h sple nic ve | 1. The spleen is enlarged, with dif-fuse, relatively increased metabolic activity.<br>2. The liver is enlarged, without ab-normal findings. | Possi-ble spleni c lym-phom a | Ap-pro xi-mat ely 2 | Following splenectomy, spleen pathology revealed suspicious pathogens within histiocytes, though no defini-tive diagnosis was made. Ap-proximately 2 months later, |

| | | | | | | | | | |
|---|---|---|---|---|---|---|---|---|---|
| | | | | | | in dilatation; bilateral cervical and axillary lymphadenopathy. | | | months | symptoms recurred; serum rK39 antibody test was positive; and prior genetic sequencing of spleen tissue suggested *Leishmania donovani* infection. |
| Nina Jancar, 2023. Portugal[12] | 38, F | History of primary hyperparathyroidism (post-parathyroidectomy) and depression (long-term treatment with escitalopram and bromazepam) | No | FUO etiology investigation | Yes | Hepatosplenomegaly; para-aortic lymphadenopathy. | Significantly increased metabolic activity was observed in the spleen and bone marrow | NR | NR | *Leishmania* amastigotes were identified in both bone marrow smears and biopsies. |
| Liuxue Wang, 2023. China[13] | 60, M | NR | NR | NR | No | Splenomegaly | 1. Splenomegaly with increased FDG uptake.<br>2. Diffusely increased FDG uptake in the bone marrow.<br>3. Concomitant bilateral pneumonia and pleural effusion. | HPS | Approximately 1 month | Recurrence of the condition following HPS chemotherapy prompted a repeat bone marrow aspiration. The bone marrow smear revealed *Leishmania* amastigotes. Serology was positive for rK39 antibodies, and peripheral blood mNGS identified *Leishmania infantum* and *L. donovani*. |
| Shipra Gulati, 2023.India[14] | 59, M | None | No | FUO etiology | No | Hepatosplenomegaly | 1. Hepatosplenomegaly with mild FDG uptake (liver size: 21.5 cm; spleen size: 11.9 cm).<br>2. Diffuse FDG uptake in the bone marrow. | Secondary HPS | Approximately | Recurrence of the condition following anti-infective and corticosteroid therapy prompted a repeat bone marrow aspiration. The smear re |

| | | | | | | | | | | |
|---|---|---|---|---|---|---|---|---|---|---|
| | | | | investigation | | | | | 5 months | vealed numerous intracellular and extracellular LD bodies accompanied by marked hemophagocytosis, suggesting VL complicated by HPS. |
| Hui Feng, 2023. China [15] | 55, M | Chronic hepatitis B | No | FUO etiology investigation | No | Hepatosplenomegaly | 1. Splenomegaly with markedly and diffusely increased FDG uptake.<br>2. Mildly and diffusely increased FDG uptake in the bone marrow. | Splenic lymphoma | Approximately 2 months | Due to the poor efficacy of HPS chemotherapy, splenectomy was performed to identify the etiology. *Leishmania* amastigotes were detected in both the spleen tissue and the third bone marrow biopsy. |
| Victor Bertolo Gomes Porto, 2022. Brazil [16] | 65, M | Advanced nodular sclerosis Hodgkin lymphoma | Yes (Advanced nodular sclerosis Hodgkin lymphoma) | NR | Yes | Generalized lymphadenopathy | 1. Multiple lymph nodes with increased glycolytic metabolism above and below the diaphragm, some coalescing into clusters.<br>2. Increased glycolytic metabolism in the spleen, liver, and bones, suggestive of a lymphoproliferative disorder. | NR | NR | Direct microscopic examination of bone marrow aspirates revealed *Leishmania* parasites. The presence was subsequently confirmed by PCR and culture of the aspirate, and the species was further identified as *Leishmania amazonensis*. Inguinal lymph node biopsy showed high-grade nodular sclerosis Hodgkin lymphoma, and immunohistochemistry was positive for *Leishmania*. |
| Antonio Giovanni Solimando, 2022. Italy [17] | 42, M | Type 1 autoimmune hepatitis | Yes (corticosteroids, immunosuppressants) | FUO etiology investigation | Yes | Splenomegaly; intra-abdominal lymphadenopathy; no hepatic abnormality. | Increased FDG uptake in the liver, spleen, and the whole bone marrow. | NR | NR | Bone marrow smear and biopsy revealed *Leishmania infantum* amastigotes; serology was positive for anti-*Leishmania* IgG antibodies. |

| | | | | | | | | | | |
|---|---|---|---|---|---|---|---|---|---|---|
| Qi Song, 2022. China [18] | 46, M | Hepatitis B virus infection | NR | FUO etiology investigation | Yes | Splenomegaly | Splenomegaly with diffusely increased FDG uptake. | NR | NR | *Leishmania* parasites were identified on bone marrow aspiration smear and detected by blood mNGS. |
| I Zaghi, 2022. Italy[19] | 59, F | Sjögren's syndrome | NR | NR | NR | Hepatosplenomegaly; aortocaval, mesenteric, and inguinal lymphadenopathy. | 1. Multiple foci of increased FDG uptake in the liver and spleen (SUVmax 10.6).<br>2. A solitary focal lesion with increased FDG uptake in a lumbar vertebra (SUVmax 10.6). | NR | NR | Biopsy of a hepatic lesion was performed. Initial pathological examination revealed no pathogens. However, peripheral blood enzyme immunoassay (EIA) and rK39 immunochromatographic test (ICT) were positive for anti-*Leishmania* IgG antibodies, and peripheral blood PCR was also positive for *Leishmania*. On re-examination of the hepatic histological specimens, *Leishmania* amastigotes were identified, and *Leishmania*-specific PCR of the specimens was positive |
| Andreas Dunzinger, 2022. Austria [20] | 72, M | Melanoma | Yes (immunotherapy for malignancy) | NR | NR | Splenomegaly | Two lesions were observed in liver segments VI and VIII, with SUVmax of 8.3 and 6.5, respectively, and a mean liver SUV of 2.6. After treatment, the PET/CT findings turned negative. | NR | Approximately 1 month | Iliac crest biopsy revealed *Leishmania* amastigotes; blood PCR was positive for *Leishmania infantum/donovani* |
| Harriet P Pinnegar, 2021. Spain [21] | 63, F | Idiopathic pulmonary fibrosis, status post left lung transplantation. | Yes | NR | Yes | Splenomegaly; paratracheal | 1. Diffuse splenic FDG uptake without focal lesions (SUVmax 6.8).<br>2. Enlarged paratracheal lymph nodes with increased FDG uptake (SUVmax 6.1 and 7.1).<br>3. Fibrosis in the right lung base. | NR | NR | Numerous *Leishmania* amastigotes observed intracellularly and extracellularly |

| | | | | | | | | | | |
|---|---|---|---|---|---|---|---|---|---|---|
| | | | | | | lymphadenopathy. | | | | |
| Harriet P Pinnegar, 2021. Spain [21] | 18, M | None | No | NR | Yes | Hepatosplenomegaly; lymphadenopathy. | 1. Splenomegaly with diffusely and heterogeneously increased FDG uptake (SUVmax 5).<br>2. Increased bone marrow FDG uptake.<br>3. Hepatomegaly (SUVmax 3.9).<br>4. Lymphadenomegaly with increased FDG uptake (SUVmax 3.4–5.6). | NR | NR | *Leishmania* amastigotes observed within macrophages and extracellularly. |
| Harriet P Pinnegar, 2021. Spain [21] | 73, M | None | No | FUO etiology investigation | No | Splenomegaly; lymphadenopathy. | 1. Mild splenomegaly with intensely and diffusely increased FDG uptake (SUVmax 14.9).<br>2. Enlarged right axillary lymph nodes with increased FDG uptake (SUVmax 17.5–18.6).<br>Follow-up PET/CT after 3 weeks of treatment showed improvement in the aforementioned lesions. | NR | NR | Bone marrow PCR was positive for *Leishmania infantum*. |
| Harriet P Pinnegar, 2021. Spain [21] | 60, M | Chronic obstructive pulmonary disease, status post bilateral lung transplantation. | Yes | NR | Yes | NR | 1. Diffusely and homogeneously increased FDG uptake in the spleen (SUVmax 4.9).<br>2. Generalized increased FDG uptake in the bone marrow (SUVmax not measured). | NR | NR | *Leishmania* amastigotes observed within bone marrow macrophages |
| Qi Shi, 2021. China [22] | NR, NR | NR | NR | NR | NR | Hepatosplenomegaly | Marked hepatosplenomegaly with diffusely increased FDG uptake in the liver (SUVmax 4.3), spleen (SUVmax 5.6), and spine (SUVmax 2.5). | NR | NR | NR |

| | | | | | | | | | | |
|---|---|---|---|---|---|---|---|---|---|---|
| Linfeng Zou, 2021. China [23] | 30, M | None | No | NR | No | Splenomegaly | 1. Splenic longitudinal diameter 30 cm (SUVmax 5.92).<br>2. Bone marrow SUVmax 4.66. | Suspected malignancy | Approximately 5 months | Bone marrow biopsy and splenectomy were performed. Pathology showed scattered plasma cell infiltration in the bone marrow, and splenic necrosis with reactive hyperplasia. A repeat bone marrow biopsy 5 months later revealed *Leishmania* parasites. |
| Dimitrios Patoulias, 2021. Greece [24] | 63, F | Rheumatoid arthritis, coronary artery disease, and hypertension. | Yes (corticosteroids, immunosuppressants) | FUO etiology investigation | No | No significant abnormalities (no splenomegaly) | Increased splenic FDG uptake without splenomegaly. | NR | NR | PET/CT findings prompted further investigation for intracellular pathogens; quantitative anti-*Leishmania* antibody testing was positive (ELISA). |
| Huiting Liu, 2021. China [25] | 18, F | NR | NR | FUO etiology investigation | Yes | Splenomegaly; lymphadenopathy. | Lymphadenopathy and splenomegaly with markedly increased FDG uptake. | NR | NR | Bone marrow smear confirmed VL. |
| Guangqiang Meng, 2020. China [26] | 26, F | NR | NR | NR | No | Hepatosplenomegaly | Spleen shows homogeneous hyper metabolism on FDG-PET. | HPS | Approximately 2 months | Peripheral blood mNGS had previously detected *Leishmania*, but it was overlooked due to the low sequence count. The condition recurred after chemotherapy for HPS. The third bone marrow smear revealed *Leishmania donovani*; peripheral blood was positive for anti-*L.* |

| | | | | | | | | | | |
|---|---|---|---|---|---|---|---|---|---|---|
| | | | | | | | | | | *donovani* IgG antibodies and *L. donovani* DNA. |
| Selda Sayın Kutlu, 2019. Turkey [27] | 49, M | None | No | FUO etiology investigation | No | Hepatosplenomegaly | Hepatosplenomegaly with diffusely increased splenic FDG uptake (SUVmax 3.63). | NR | NR | *Leishmania* amastigotes were identified in the second bone marrow aspirate. |
| Selda Sayın Kutlu, 2019. Turkey [27] | 75, F | Diabetes mellitus, hypertension. | No | FUO etiology investigation | No | Hepatosplenomegaly; intra-abdominal lymphadenopathy. | 1. Hepatosplenomegaly with diffusely mildly increased splenic FDG uptake (SUVmax 1.77) and multiple superimposed low-density nodules.<br>2. Diffusely increased FDG uptake in the bone marrow of the entire skeleton (SUVmax 2.18–3.05). | NR | NR | Following splenectomy, splenic histopathological examination revealed no *Leishmania* amastigotes, whereas the immunofluorescent antibody test for anti-*Leishmania* IgG was positive. |
| Iratxe Martinez de Narvajas, 2019. Spain [28] | 55, M | Hypertension | No | FUO etiology investigation | No | Splenomegaly | Splenic FDG uptake (SUVmax 13.26) and diffusely increased FDG uptake in the liver and bone marrow. | NR | NR | Diagnostic liver biopsy revealed *Leishmania* amastigotes within macrophages; PCR testing of the biopsy tissue was positive for *Leishmania infantum*. |
| I. Machelart, 2019. France [29] | 76, M | Localized prostate cancer, skin cancer, both in complete remission. | No | NR | No | Hepatosplenomegaly | 1. Bilateral small inguinal lymph nodes (<1 cm) with increased FDG uptake.<br>2. Hypermetabolic hepatosplenomegaly. | HPS | NR | The disease recurred following chemotherapy for HPS. The patient underwent splenectomy, and histopathological examination of the spleen revealed histiocytes containing numerous *Leishmania* amastigotes. PCR testing of serum and splenic tissue |

| | | | | | | | | | | |
|---|---|---|---|---|---|---|---|---|---|---|
| | | | | | | | | | | samples was positive for *Leishmania infantum*. Retrospective analysis showed that PCR of bone marrow aspirate was also positive. |
| Francesca Rinaldi, 2019. Italy[30] | 32, M | Type 1 diabetes mellitus and Hashimoto thyroiditis | No | NR | No | Splenomegaly | Diffusely increased FDG uptake in the spleen with superimposed multifocal hypermetabolic foci. | Suspected hematologic disorders | NR | PCR testing of bone marrow and peripheral blood was positive for *Leishmania*. |
| Lucia Zanoni, 2019. Italy[31] ‡ | Median age was 60 years (range 24–82), The majority were males | NR | No (21 cases were immunocompetent) ‡ | NR | NR | NR | Six cases showed isolated splenic FDG uptake; two cases showed splenic and lymph node FDG uptake; seven cases showed splenic and bone marrow FDG uptake; four cases showed splenic, bone marrow, and lymph node FDG uptake; one case showed splenic, bone marrow, and lung FDG uptake; one case showed bone marrow and lymph node FDG uptake; and two cases showed isolated lymph node FDG uptake. Splenic FDG uptake exhibited two patterns: a diffuse pattern (13/20 cases; mean spleen SUVmax 7.3 ± 4.2; range 4.0–14.1) and a focal pattern on a diffuse background (7/20 cases; mean SUVmax 12.6 ± 4.5; range 9.5–20.5). The mean bone marrow SUVmax was 4.0 ± 1.3 (range 2.8–6.0). | NR | NR | Microbiological diagnosis of VL was confirmed in all patients by serological and molecular methods |
| Verena Schwetz, 2018. Austria [32] | 60, M | Type 2 diabetes mellitus, hypothyroidism. | No | FUO etiology | Yes | Splenomegaly | 1. Uniformly increased FDG uptake in the spleen.<br>2. Uniformly increased FDG uptake in the central and peripheral bone marrow. | NR | NR | Bone marrow biopsy revealed microorganisms within macrophages, and PCR testing of peripheral blood and bone samples was positive for *Leishmania donovani*. |

| | | | | | | | | | |
|---|---|---|---|---|---|---|---|---|---|
| | | | | in-ves-tiga-tion | | | | | |
| E.B.D. Molendijk, 2018. Netherlands[33] | 63, M | None | No | FUO etiol-ogy in-ves-tiga-tion | No | Sple-no-meg-aly | Splenomegaly with intense FDG up-take. | Considering the relatively high likelihood of malignant splenic lymphoma | NR | Spleen and bone marrow bi-opsies were performed. The spleen biopsy revealed *Leish-mania* parasites, while the bone marrow biopsy showed no parasites. PCR testing of peripheral blood was positive for *Leishmania infantum*. |
| P. Tatarelli, 2018. Italy[34] | 24, M | Nodular sclerosis Hodgkin lymphoma, status post hematopoi-etic cell transplanta-tion. | Yes (chemoradi-other-apy for malig-nancy, hema-topoi-etic cell trans-planta-tion) | NR | Yes | Sple-no-meg-aly; multi-ple in-tra-ab-dominal lym-phad-e-nopa-thy. | 1. Multiple enlarged intra-abdominal lymph nodes with increased FDG up-take (SUVmax 8).<br>2. Splenomegaly (diameter 16.5 cm) with diffusely increased FDG uptake within the splenic parenchyma (SUVmax 3.15).<br>3. Diffusely increased FDG uptake in the bone marrow.<br>Post-treatment PET/CT demonstrated no abnormal FDG uptake. | Considering a case consistent with Hodgkin's lymphoma, possibly accompanied by splenic involvement. | Ap-pro xi-mat ely 1 month | Bone marrow aspiration re-vealed *Leishma-nia* amastigotes. PCR testing of peripheral blood was posi-tive, and anti-*Leishmania* an-tibodies were detected in se-rum. |

| | | | | | | | | | | |
|---|---|---|---|---|---|---|---|---|---|---|
| Anna Eichen berger, 2017. Switzer land[35] | 80, M | Hypertensio n, type 2 diabetes m ellitus, abd ominal aort ic aneurys m surgery and previo usly treated Q fever, as well as non-specifi c inflamma tory diseas es of the l iver and ki dneys. | Yes (corti-coster-oids, immu-nosup-pres-sants) | As-sess-ment for Dis-ease Dis-sem-ina-tion and Se-ver-ity Fol-low-ing VL Di-ag-nosis | NR | Hepat osple-no-meg-aly | Diffusely increased FDG uptake in the colon, the enlarged spleen (18 cm), the liver, the peritoneal lymph nodes, and the basal segments of the lungs. | Not a pplica ble. | Not ap-pli-ca-ble. | Diagnosis of VL was confi rmed by colon histopatholo gy showing amastigotes, ser ological antibody detection, and PCR. |
| Gilles Darcis, 2017. Belgiu m [36] | 52, F | Rheumatoid arthritis complicated by vascu-litis, cryo-globuline-mia, classi-cal Hodgkin lymphoma, status post splenec-tomy, and VL (diag-nosed 2 years ago) | Yes (corti-coster-oids, immu-nosup-pres-sants, chemo radio-ther-apy for malig-nancy) | NR | Yes | Gener alized lymph adeno pathy (cervi cal, s ubclav icular, supra diaphr agmat ic, an d infr adiaph ragma tic). | Rapidly progressive generalized lym-phadenopathy with increased FDG uptake. After treatment, follow-up PET/CT showed regression of the le-sions. | NR | NR | Lymph node biopsy revealed extensive infiltration by mac-rophages containing LD bod-ies, suggestive of VL recur-rence. |
| Ritesh Prajapa | 52, M | NR | No | FUO | No | Hepat osplen | 1. Hepatosplenomegaly with chronic liver disease. | NR | NR | Liver biopsy revealed LD bodies within histiocytes, |

| | | | | | | | | | |
|---|---|---|---|---|---|---|---|---|---|
| ti, 2016. India[37] | | | | etiology investigation | | omegaly | 2. Diffusely increased FDG uptake in the bone marrow.<br>3. Small amount of ascites. | | | with elevated anti-*Leishmania* IgG. |
| Leonardo Chianura, 2014. Italy[38] | 53, F | End-stage renal disease (ESRD) of unknown etiology, status post renal transplantation. | Yes (corticosteroids, immunosuppressants) | FUO etiology investigation | Yes | Splenomegaly | Diffusely increased FDG uptake in the enlarged spleen. | NR | 10 days | Bone marrow smear revealed numerous *Leishmania* amastigotes within macrophages. |
| Leonardo Chianura, 2014. Italy[38] | 45, M | HIV infection, type 1 diabetes mellitus. | Yes (HIV infection) | FUO etiology investigation | Yes | Splenomegaly | 1. Splenomegaly with diffusely increased FDG uptake.<br>2. Diffuse FDG uptake in the spine. | NR | NR | Bone marrow aspiration revealed *Leishmania* amastigotes. |
| Thibault Comont, 2014. France [39] | 64, M | Massive lymphadenopathy (Rosai-Dorfman disease) | NR | NR | No | Hepatosplenomegaly; submandibular and intra-abdominal lymphadenopathy. | Submandibular and abdominal lymphadenopathy, hepatomegaly, and splenomegaly with marked hypermetabolism.<br>Follow-up PET/CT after 2 months of treatment demonstrated complete resolution of all hypermetabolic foci. | NR | NR | Serological testing for *Leishmania* was positive, and PCR targeting the *Leishmania* complex on blood and bone marrow aspirates was also positive. |
| Jordi Fuertes, 2014. Spain [40] | 44, F | Gaucher disease and secondary portal hypertension | NR | FUO etiology | NR | Left internal mammary | The initial PET/CT demonstrated increased FDG uptake and thickening of the left visceral and parietal pleura, left internal mammary lymphadenopathy with focally increased | NR | NR | The patient underwent splenectomy and liver biopsy. Histopathological examination revealed subcapsular splenic infarcts filled |

| | | | | | | | | | | |
|---|---|---|---|---|---|---|---|---|---|---|
| | | | | investigation | | lymphadenopathy. | FDG uptake, and inflammatory changes in the ascending colon (observed on both PET and CT); however, the cause of fever remained inconclusive. A follow-up PET/CT revealed multiple subcapsular hypodense splenic lesions with increased FDG uptake, and splenectomy was recommended. | | | with *Leishmania* amastigotes, which were also observed within hepatocytes. |
| Valentina Gallina, 2014. Italy[41] | 72, M | Renal cell carcinoma cured by nephrectomy | No | Potential Biopsy Site Localization | No | Splenomegaly | 1. Splenomegaly with pathological accumulation of radiopharmaceuticals.<br>2. Mild and homogeneous FDG uptake in the bone marrow. | NR | NR | May-Grünwald–Giemsa staining was performed on the bone marrow samples, and microscopic examination revealed abundant *Leishmania* amastigotes. Serological examination revealed an anti-*Leishmania* IgG titer of 1:320. |
| David Guard, 2014. Australia [42] | 48, M | None | No | NR | No | Hepatosplenomegaly | Abnormal splenic uptake. | NR | NR | Following splenectomy, histopathological examination revealed abundant amastigotes. |
| Guoqun Mao, 2014. China [43] | 45, M | NR | NR | FUO etiology investigation | NR | Splenomegaly | Increased 18F-FDG metabolism in the spleen. | Suspected lymphoma | NR | Serological testing for anti-*Leishmania* antibodies was positive. |
| D.H. van Raalte, 2014. Netherlands[44] | 69, M | Diabetes mellitus, myocardial infarction complicated by cardiac arrest, and hereditary | No | NR | No | Splenomegaly | Increased uptake in the bone marrow and spleen. | NR | NR | On re-examination of the bone marrow specimen, *Leishmania* amastigotes were identified, and *Leishmania infantum* infection was confirmed by direct agglutination test and PCR. |

| | | | | | | | | | | |
|---|---|---|---|---|---|---|---|---|---|---|
| | | elliptocytosis | | | | | | | | |
| M. Murri, 2014. Italy[45] | 61, M | Polycythemia vera, hemolytic anemia. | Yes (corticosteroids) | FUO etiology investigation | No | Splenomegaly | Hypermetabolic activity in the spleen and bone marrow. | NR | NR | Repeat bone marrow biopsy revealed numerous macrophages with intracellular LD bodies. |
| Glenna M. Gibson, 2014. Australia[46] | 48, M | None | No | FUO etiology investigation | No | Hepatosplenomegaly | 1. Marked splenomegaly with extensive, nodular areas of moderate to marked FDG uptake (diffuse FDG uptake with superimposed focal/nodular regions).<br>2. Diffuse FDG uptake in the bone marrow. | NR | NR | Following splenectomy, histopathological examination of the spleen revealed *Leishmania* amastigotes, which were confirmed by PCR as *Leishmania donovani*. |
| Georg Evers, 2013. Germany [47] | 57, M | Type 1 diabetes mellitus, hypertension, allergic rhinitis, and Lyme disease treated at a hospital 5 years ago. | No | FUO etiology investigation | No | Hepatosplenomegaly | Splenomegaly with increased metabolic activity (SUV 6.8). | Preliminary diagnosis: splenic marginal zone lymphoma with autoimmune hemolytic anemia. | Approximately 2 months | Splenectomy was performed, but histopathological evaluation of the spleen revealed no evidence of malignancy. Two months later, a second bone marrow aspiration demonstrated numerous macrophages and monocytes filled with intracellular inclusion bodies of *Leishmania infantum*. PCR and serological testing confirmed the diagnosis of VL. Histopathological re-examination of the previous splenic specimen showed scattered inclusion bodies of *Leishmania infantum* within macrophages in the red pulp. |
| Catherine E Berry, 2013. Austral | 69, F | Rheumatoid arthritis, atrial fibrillation, coronary artery | Yes | FUO etiology | No | Splenomegaly | Markedly increased FDG uptake in the spleen. | NR | NR | Following splenectomy, histopathological examination revealed abundant amastigotes within macrophages. Re-examination of |

| | | | | | | | | | | |
|---|---|---|---|---|---|---|---|---|---|---|
| ia [48] | | disease, hypertension, and pulmonary infiltrates presumed to be rheumatoid nodules (stable on CT for 3 years) | (immunosuppressants) | investigation | | | | | | the bone marrow biopsy specimen likewise detected the parasites. PCR analysis of splenic tissue confirmed *Leishmania infantum*. A retrospective review of the initial bone marrow specimen identified a single macrophage containing amastigotes. |
| Lin Wu, 2013. China [49] | 19, M | NR | NR | NR | No | Hepatosplenomegaly | 1. Diffusely increased FDG uptake in both the central and peripheral bone marrow.<br>2. Hepatomegaly and splenomegaly with slightly increased splenic FDG uptake. | NR | NR | The second bone marrow smear revealed *Leishmania donovani* amastigotes. |
| P.G. Kyrtatos,2013. UK[50] | 44, M | HIV-1 infection | Yes (HIV infection) | Suspected Lymphoma | No | Splenomegaly | 1. Multiple foci of increased FDG uptake in the spleen and retroperitoneal lymph nodes.<br>2. Diffuse FDG uptake in the bone marrow.<br>Follow-up PET/CT at one month post-treatment showed improvement. | NR | NR | Serological testing (ELISA) and PCR testing of peripheral blood for *Leishmania* were both positive. PCR testing of the bone marrow was also positive. |
| Claudia Ravaglia, 2013. Italy[51] | 42, F | Sarcoidosis | Yes (corticosteroids, immunosuppressants) | NR | Yes | Hepatosplenomegaly; mediastinal | Multiple foci of increased FDG uptake in the liver, spleen, thoracoabdominal lymph nodes, third thoracic vertebra, and bilateral nodular infiltrates. | NR | NR | Bone marrow biopsy revealed *Leishmania* parasites. |

| | | | | | | | | | | |
|---|---|---|---|---|---|---|---|---|---|---|
| | | | | | | lymphadenopathy. | | | | |
| C Martin, 2013. Belgium[52] | NR, NR | HIV infection | Yes (HIV infection) | FUO etiology investigation | Yes | NR | Increased FDG uptake in cervical lymph nodes (SUVmax 2.9). | NR | NR | Bone marrow aspiration was performed because of abnormal PET/CT findings and led to the diagnosis of VL. |
| Ali Fuat Yapar, 2011. Turkey[53] | 51, F | NR | NR | Assessment of Nodal Metabolic Activity for Biopsy Targeting | No | Splenomegaly; splenic hilar lymphadenopathy. | 1. Splenomegaly with diffusely increased FDG uptake (SUVmax 5.3) 2. Splenic hilar lymph nodes with diffusely increased FDG uptake (SUVmax 4); mildly increased FDG uptake in lymph nodes anterior to the vena cava (SUVmax 3.8) 3. Diffusely increased FDG uptake in the bone marrow. | NR | NR | The patient underwent splenectomy, and pathological analysis of the splenic tissue revealed *Leishmania* amastigotes. Bone marrow biopsy showed no significant pathological changes, with only mild cellular proliferation. |
| Brett P. Bielory, 2011. America[54] | 39, M | HIV infection | Yes (HIV infection) | NR | NR | Hepatosplenomegaly | Increased FDG uptake in the lateral conjunctiva of the right eye, liver, spleen, and axillary, mediastinal, mesenteric, pelvic, and cervical lymph nodes. | NR | NR | Orbital tissue biopsy revealed *Leishmania* amastigotes, which were identified as *L. donovani chagasi*, and liver biopsy confirmed the diagnosis of VL. |
| M. Casadevall, 2010. France[55] | 19, M | NR | NR | FUO etiology investigation | No | NR | Isolated, pseudonodular, and heterogeneous increased FDG uptake in the spleen. | Suspected splenic neoplasm | NR | Splenectomy was planned; however, the indirect immunofluorescence serological test for *Leishmania infantum* returned a strongly positive result, and both blood and bone marrow PCR tests detected *Leishmania* spp. D |

| | | | | | | | | | | |
|---|---|---|---|---|---|---|---|---|---|---|
| | | | | | | | | | | NA. |
| Andrea Lupi, 2006. Italy[56] | 48, M | NR | NR | FUO etiology investigation | Yes | NR | 1. Markedly increased FDG uptake in the spleen, with a patchy and granular pattern.<br>2. Scattered and diffuse areas of increased FDG uptake in the liver.<br>3. Scattered and diffuse increased FDG uptake in the skeleton, including all vertebral bodies, the sternum, and, to a lesser extent, the sacrum. | NR | NR | Microscopic examination of a bone marrow smear revealed *Leishmania* amastigotes. |

**Note:** FDG, $^{18}$F-fluorodeoxyglucose; SUV, standardized uptake value; SUVmax, maximum standardized uptake value; LD bodies, Leishman–Donovan bodies; HPS, hemophagocytic syndrome; ELISA, enzyme-linked immunosorbent assay; PCR, polymerase chain reaction; NR, not reported; M, male; F, female； FUO, fever of unknown origin; PET/CT, $^{18}$F-fluorodeoxyglucose positron emission tomography/computed tomography; VL, visceral leishmaniasis; BM, bone marrow; mNGS, metagenomic next-generation sequencing. ‡ In Zanoni et al. [31] study, the text states 21 PET/CT-positive patients (all immunocompetent), whereas the detailed organ involvement table lists positive combinations summing to 23. This row includes all 23 positive records from the published table to preserve the full anatomical data. In the immune-status subgroup analysis in the main text, only the 21 positive patients with clearly linked immune status were included; the remaining 2 positive records, for which immune status could not be independently confirmed, were not assigned to any immune category (see the Main Findings section for handling of the numerical difference).

**Table S1-B.** Summary of detailed information for all cases identified through the literature review — cases classified as “Explicitly Negative” on FDG-PET/CT

| **Author(s), Year, Region of consultation** | **Age (years), Sex** | **Underlying disease** | **Immunosuppression** | **Indication for PET/CT** | **Initial BM smear/biopsy: Leishmania am** | **General Imaging and Physical Examination Findings for Hepatomegaly, Splenom** | **PET/CT findings** | **Diagnosis after PET/CT** | **Time from PET/CT to VL confirmation** | **Process and methods for VL confirmation after PET/CT** |
|---|---|---|---|---|---|---|---|---|---|---|

| | | | | | | | | | |
|---|---|---|---|---|---|---|---|---|---|
| | | | | | **astigotes detected** | **egaly, and Lymphadenopathy** | | | |
| Vinny Ea, 2024. Australia[57] | 72, M | Type 2 diabetes mellitus | No | NR | No | Splenomegaly | No abnormal metabolic activity was observed. | Autoimmune hepatitis | NR | The response to autoimmune hepatitis treatment was poor. A second bone marrow aspiration revealed *Leishmania* amastigotes, and PCR was positive for the *Leishmania donovani* complex. |
| Lucia Zanoni, 2019. Italy[31] | Median age was 60 years (range 24–82), The majority were males | NR | Yes | NR | NR | NR | Two cases demonstrated no abnormal FDG uptake. | NR | NR | Microbiological diagnosis of VL was confirmed in all patients by serological and molecular methods. |
| Yuling Pan, 2014. China | 34, M | NR | NR | NR | No | Splenomegaly | No significant hypermetabolism. | NR | NR | *Leishmania* parasites were identified in a second bone marrow smear. |

| | | | | | | | | | | |
|---|---|---|---|---|---|---|---|---|---|---|
| [58] | | | | | | | | | | |
| Fangyi Fan, 2012. China [59] | 24, M | NR | NR | NR | No | Sple-no-meg-aly | Bilateral small pleural effusions without increased FDG uptake; no other abnormalities identified. | HPS | Approximately 1.5 months | Following disease recurrence after HPS chemotherapy, the second bone marrow aspiration smear revealed abundant LD bodies, and the rK39 antibody test was positive. |

**Note:** PET/CT,$^{18}$F-fluorodeoxyglucose positron emission tomography/computed tomography; VL, visceral leishmaniasis; BM, bone marrow; PCR, polymerase chain reaction; FDG, $^{18}$F-fluorodeoxyglucose; LD bodies, Leishman–Donovan bodies; HPS, hemophagocytic syndrome; NR, not reported; M, male.

**Table S1-C.** Summary of detailed information for all cases identified through the literature review — cases classified as "Non-evaluable" on FDG-PET/CT

| **Author(s), Year, Region of consultation** | **Age (years), Sex** | **Underlying disease** | **Immunosuppression** | **Indication for PET/CT** | **Initial BM smear/biopsy: Leishmania amastigotes detected** | **General Imaging and Physical Examination Findings for Hepatomegaly, Splenomegaly, and Lymphadenopathy** | **PET/CT findings** | **Diagnosis after PET/CT** | **Time from PET/CT to VL confirmation** | **Process and methods for VL confirmation after PET/CT** |
|---|---|---|---|---|---|---|---|---|---|---|
| Illiasul K Iba | middl | Type 2 diabetes | No | NR | Yes | NR | No evidence of infection; diffuse reduction in trunk and limb muscle | Suspecte | NR | Peripheral blood and bone marrow smears revealed L |

| | | | | | | | | | | |
|---|---|---|---|---|---|---|---|---|---|---|
| d, 2025. India[60] | e-aged, M | mellitus | | | | | volume. | d HPS | | D bodies. Anti-RK39 antibody testing and *Leishmania* PCR were positive, confirming the pathogen as *Leishmania donovani*. |
| Hongmei Wang, 2025. China [61] | 31, M | None | No | FUO etiology investigation | No | Splenomegaly | Splenomegaly and lymph node hyperplasia at multiple sites. | Suspected hematologic malignancy | NR | mNGS of peripheral blood detected *Leishmania infantum* and *Leishmania donovani*. |
| Ilir Tolaj, 2023. Kosovo [62] | 62, M | NR | NR | NR | No | Hepatosplenomegaly | Prominent splenic involvement with concurrent hepatic and bone marrow involvement. | NR | Approximately 6 months | Following refusal of diagnostic splenectomy and unsuccessful empiric antibiotic treatment, a repeat bone marrow aspiration six months later demonstrated *Leishmania infantum* amastigotes on smear, with positive *Leishmania* IgG serology. |
| Ying Liu, 2023. China [63] | 65, M | None | No | NR | Yes | Splenomegaly | 1. Splenic lymphoma suspected.<br>2. Bilateral pulmonary emphysema and scattered inflammatory changes.<br>3. Non-specific lymphadenopathy in the mediastinum and bilateral hilar regions. | NR | NR | Pathological examination of the splenic aspirate revealed suspected *Leishmania* amastigotes, whereas bone marrow smear definitively demonstrated the parasites. The blood rK39 antigen test was positive, and *Leishmania* PCR also returned a positive result. |
| Shaowei Huang, 2023. China [64] | 49, M | Chronic bronchitis | No | NR | No | Splenomegaly; right inguinal lymphadenopathy. | Active foci of bone metabolism in the bilateral knees, with chronic inflammatory changes; no other abnormalities were observed on bone metabolism imaging of the rest of the body, suggesting chronic knee arthritis. | NR | NR | Blood mNGS was positive for *Leishmania infantum*. |

| | | | | | | | | | | |
|---|---|---|---|---|---|---|---|---|---|---|
| GuoQiang Zhang, 2020. China [65] | 25, M | Chronic hepatitis B virus infection, left indirect inguinal hernia. | No | NR | No | Hepatosplenomegaly; supraclavicular and inguinal lymphadenopathy. | Marked hepatosplenomegaly with portal vein dilatation (14 mm) and mild ascites, without evidence of malignant neoplasm. | NR | NR | The third bone marrow smear and biopsy revealed *Leishmania* amastigotes extracellularly and within macrophages; a concurrently tested plasma sample was positive for anti-*Leishmania donovani* antibodies. |
| Feifei Lei, 2017. China [66] | 46, M | None | No | NR | No | Splenomegaly | Splenic mass: Lymphoma should be the first consideration. Splenectomy is recommended for pathological examination. | Lymphoma | Approximately 1 year | The patient underwent splenectomy, and pathological examination of the spleen revealed splenic infarction. Approximately one year later, the rK39 antibody test was positive. |
| Yongqiang Wu, 2017. China [67] | 60, M | None | No | NR | No | Splenomegaly | No evidence of tumor. | HPS | Approximately 1 month | Chemotherapy for HPS showed poor efficacy. The third bone marrow smear revealed *Leishmania* amastigotes. |
| F. Molinaro, 2014. Italy[68] | 61, F | Oligoarthritis | Yes (immunosuppressants) | FUO etiology investigation | No | Splenomegaly | PET/CT findings not specified. | NR | NR | One month after empirical VL treatment, the anti-*Leishmania infantum* antibody test was positive. |
| Maja Ø. Vase, 2012. Denmark [69] | 60, M | Asthma, hypereosinophilia, and primary splenic marginal zone ly | Yes (immunosuppressants) | NR | No | Splenomegaly | No evidence of lymphoma. | NR | NR | Repeat bone marrow biopsy revealed *Leishmania* amastigotes. Retrospective review of all previous biopsy specimens (2 bone marrow biopsies, 1 liver biopsy, |

| | | | | | | | | | | |
|---|---|---|---|---|---|---|---|---|---|---|
| | | mphoma treated with splenectomy. | | | | | | | | and 1 spleen biopsy) showed the same organisms in all specimens. |
| (CDC) Centers for Disease Control & Prevention, 2004. America [70] | 31, M | None | No | NR | No | Hepatosplenomegaly | Splenic findings suspicious for lymphoma. | NR | Approximately 1 month | Re-examination of the liver biopsy specimen revealed *Leishmania* parasites. |

**Note**: FDG,[18]F-fluorodeoxyglucose; PET/CT, 18F-fluorodeoxyglucose positron emission tomography/computed tomography; VL, visceral leishmaniasis; BM, bone marrow; HPS, hemophagocytic syndrome; NR, not reported; LD bodies, Leishman–Donovan bodies; PCR, polymerase chain reaction; FUO, fever of unknown origin; mNGS, metagenomic next-generation sequencing; M, male; F, female.

## References


1. Ceylan S, Mert A: A Case of Kala-azar Diagnosed via PET/CT and Confirmed by Splenectomy. *Medical Science and Disco very* 2025, 12(4):136-140. doi: 10.36472/msd.v12i4.1274.
2. Arzel M, Bellini C, Milowich D, Nabergoj M: Visceral Leishmaniasis Mimicking Aggressive Lymphoma. *Cureus* 2025, 17 (9):e92843. doi: 10.7759/cureus.92843.
3. Gestels N, Bottieau E, De Vooght A, van Ierssel S, Libbrecht S, Driessen A, Francque S, Vanwolleghem T, Kwanten WJ: An unusual case of acute cholestatic hepatitis: visceral leishmaniasis with secondary haemophagocytic lymphohistiocyto sis. *Acta Gastroenterol Belg* 2025, 88(1):78-84. doi: 10.51821/88.1.13361.
4. Guo Q, Liu X: FDG PET/CT enables splenectomy avoidance in visceral leishmaniasis-associated hemophagocytic lympho histiocytosis: A case report. *Asian Journal of Surgery* 2025, 48(11):7253-7255. doi: 10.1016/j.asjsur.2025.05.179.
5. Decaestecker A, Daniel A, Malbranque Z, Wayolle N, Ydee A: Crescentic glomerulonephritis associated with myelodyspl atic syndrome and visceral leishmaniasis. *Journal of Nephrology* 2025, 38(7):1999-2004. doi: 10.1007/s40620-025-02329-y.
6. Battino N, Pisano A: Visceral leishmaniasis and lymphoma: a rare and dangerous couple. *Italian Journal of Medicine* 2025, 19(1). doi: 10.4081/itjm.2025.1843.
7. Alghaythi S, Almuhaidb A, Al-Bawardy B: Double Trouble: Leishmaniasis and Cytomegalovirus Colitis in an Immunoco mpromised Host. *ACG Case Reports Journal* 2025, 12(5). doi: 10.14309/crj.0000000000001687.
8. Kumar Vaitheeswaran K, Gupta BK, Krishnan G R, Soneja M, Vikram NK, Baitha U, Singh A, Wig N, Azam M, Singh R *et al*: Neuro-leishmaniasis with cauda equina syndrome and cranial nerve palsy: a rare manifestation of recurrent aty pical visceral leishmaniasis. *BMC Infectious Diseases* 2024, 24(1). doi: 10.1186/s12879-024-10082-z.
9. Chang P, Al-Shyoukh A, Grewal H, Burke V: An Uncommon Cause of a Common Finding: Mediastinal Lymphadenopa thy Secondary to Leishmaniasis. *Am J Respir Crit Care Med* 2024, 209:A7508. doi: 10.1164/ajrccm-conference.2024.209.1_me etingabstracts.a7508.
10. Banegas DE, Moioli A, Santoni E, Tagliavini E, Quaglia FM, Bernardelli A, Visco C: Visceral Leishmaniasis Following A +AVD Treatment in a Patient with Classical Hodgkin's Lymphoma: A Case Report and Review of the Literature. *Journal of clinical medicine* 2024, 13(19). doi: 10.3390/jcm13195756.
11. Yang M, Xiang B, Wei T, Chi Ye, Li J: Visceral leishmaniasis complicated by myelofibrosis: a case report *Chinese Journal of Parasitology and Parasitic Diseases* 2024, 42(05):684-687. doi: 10.12140/j.issn.1000-7423.2024.05.021.
12. Jancar N, Sousa Goncalves F, Duro J, Pinto I, Oliveira T, Aguiar P: Kala-Azar: A Case Report. *Cureus* 2023, 15(2):e34864. doi: 10.7759/cureus.34864.
13. Wang L, Hu M, Wu X, Ma L, Yang H: Case Report: Diagnosis and Treatment of Two Clinical Cases of Visceral Leishm aniasis-Related Hemophagocytic Lymphohistiocytosis. *Am J Trop Med Hyg* 2023, 109(2):296-300. doi: 10.4269/ajtmh.22-0776.
14. Gulati S, Dessai R, Patnaik NK, Chawla K: Visceral leishmaniasis escaping the diagnosis and withstanding treatment in a case of recurrent pyrexia. *International Journal of Research in Medical Sciences* 2023, 11(9):3467-3470. doi: 10.18203/2320-60 12.ijrms20232812.
15. Feng H, Dai W: Case Report: FDG-PET/CT findings in co-infection of visceral leishmaniasis and chronic hepatitis B. *Fro ntiers in Cellular and Infection Microbiology* 2023, 13. doi: 10.3389/fcimb.2023.1175897.
16. Porto VBG, Carvalho LB, Buzo BF, Litvoc MN, Santos ACS, Rocci RA, Soares SRC, Zampieri RA, Duarte MIS, Lindoso JAL: Visceral leishmaniasis caused by Leishmania (Leishmania) amazonensis associated with Hodgkin's lymphoma. *Rev Inst Med Trop Sao Paulo* 2022, 64:e51. doi: 10.1590/S1678-9946202264051.
17. Solimando AG, Coniglio G, Desantis V, Lauletta G, Bavaro DF, Diella L, Cirulli A, Iodice G, Santoro P, Cicco S *et al*: A Challenging Case of Visceral Leishmaniasis. *Reports* 2022, 5(2). doi: 10.3390/reports5020023.
18. Song Q, Sheng S, Wang Y, Shao J, Li T: Investigation and analysis of a kala-azar case in Wuxi City. *Jiangsu Journal of P reventive Medicine* 2022, 33(04):459-460. doi: 10.13668/j.issn.1006-9070.2022.04.030.
19. Zaghi I, Ielasi L, Stagni B, Cascavilla A, Ferri S, Ambrosini V, Riefolo M, Varani S: A case of Leishmania infection with focal splanchnic involvement without systemic symptoms: a potential anti-inflammatory role for vitamin D. *Acta Clinica Belgica* 2022, 78(4):321-324. doi: 10.1080/17843286.2022.2145686.
20. Andreas Dunzinger1, Carina Datinger1, Almute Loidl1, Bernhard Walcherberger1, Roland Andreas, Lengauer1 NMaRP: Visceral Hepatic Leishmaniasis in a Melanoma Patient in FDG-PET. *Curr Med Imaging* 2022, 18(4):425-428. doi: 10.2174/15 73405617666210714122602. PMID: 34264187.
21. Pinnegar HP, Sánchez-Montalvá A, Barios Profitos M, Bosch-Nicolau P, Salvador F, Molina I: Utility of Fluorine-18 Fluo rodeoxyglucose Positron Emission Tomography/Computed Tomography in Patients with Visceral Leishmaniasis: Case Re port and Literature Review. *The American Journal of Tropical Medicine and Hygiene* 2021, 104(3):934-944. doi: 10.4269/ajtmh. 19-0858.
22. Shi Q, Huang M, Li X, Zheng X, Wang F, Zou Y, Wang L, Jia J: Clinical and laboratory characteristics of hemophagocy tic lymphohistiocytosis induced by Leishmania infantum infection. *PLOS Neglected Tropical Diseases* 2021, 15(11). doi: 10.1 371/journal.pntd.0009944.
23. Zou L, Chen G, Zhou Y, Ye W, Wen Y, Chen L, Li X: Continuous hypergammaglobulinemia and proteinuria after the r ecovery of the visceral Leishmaniasis: a case report. *BMC Infectious Diseases* 2021, 21(1). doi: 10.1186/s12879-021-05819-z.
24. Patoulias D, Papachristou S, Gouridou E, Stamou D, Chissan S, Spyridon B: A Patient with Rheumatoid Arthritis under

Methotrexate and Etanercept Treatment Presenting with Fever and Pancytopenia: An Unexpected Guest. *Mediterranean Journal of Rheumatology* 2021, 32(2). doi: 10.31138/mjr.32.2.160.

25. Liu H, Fan H, Huang X, Jiao Y: The clinical characteristics and outcomes of patients with fever of unknown origin caused by parasitic infection. *Medicine* 2021, 100(16). doi: 10.1097/md.0000000000025538.
26. Meng G, Wang J, Wu L, Wang Z: Visceral leishmaniasis complicated by hemophagocytic lymphohistiocytosis: a case report. *Chinese Journal of Infection and Chemotherapy 2020* 2020, 20(06):689-691. doi: 10.16718/j.1009-7708.2020.06.019.
27. Sayin Kutlu S, Kutlu M, Herek D, Dirim Erdogan D, Ozdemir K, Sen Turk N, Yaylali O, Hacıoglu S, Turgut H: Multiple Nodular Lesions in Spleen and Liver in Visceral Leishmaniasis. *Infectious Diseases and Clinical Microbiology* 2019, 1(2):70-77. doi: 10.36519/idcm.2019.19011.
28. Martinez de Narvajas I, Diaz A, Bassegoda O, Carpio A, Fuster C, Valls ME, Alvarez-Martinez MJ, Garcia-Vidal C, Soriano A, Martinez JA *et al*: Acute liver failure due to visceral leishmaniasis in Barcelona: a case report. *BMC Infect Dis* 2019, 19(1):874-879. doi: 10.1186/s12879-019-4553-7.
29. Machelart I, Lapoirie J, Viallard JF, Mirabel M, Lazaro E, Greib C, Rivière E: Visceral leishmaniasis with hemophagocytic lymphohistiocytosis in an immunocompetent adult. *Médecine et Maladies Infectieuses* 2019, 49(7):548-550. doi: 10.1016/j.medmal.2019.07.006.
30. Rinaldi F, Giachè S, Spinicci M, Corsi P, Ambu S, Gianfaldoni G, Rigacci L, Arena U, Bartoloni A, Zammarchi L: Focal spleen lesions in visceral leishmaniasis, a neglected manifestation of a neglected disease: report of three cases and systematic review of literature. *Infection* 2019, 47(4):507-518. doi: 10.1007/s15010-019-01279-5.
31. Zanoni L, Varani S, Attard L, Morigi JJ, Vanino E, Ortalli M, Fonti C, Viale P, Re MC, Fanti S *et al*: 18F-FDG PET/CT in visceral leishmaniasis: uptake patterns in the context of a multiannual outbreak in Northern Italy. *Annals of Nuclear Medicine* 2019, 33(9):716-723. doi: 10.1007/s12149-019-01381-6.
32. Schwetz V, Trummer C, Friedl C, Beham-Schmid C, Kulnik R, Wolfler A, Horvath K, Wunsch S, Prattes J, Zollner-Schwetz I *et al*: Visceral leishmaniasis in a patient with diabetes mellitus type 2 and discrete bicytopenia. *Clin Case Rep* 2018, 6(1):78-81. doi: 10.1002/ccr3.1259.
33. Delfos, N.M.; den Hartog, W.C.E.; Anten, S. Koorts uit onverwachte hoek. *Tijdschr. Infect.* 2018, 14, 188–193
34. Tatarelli P, Fornaro G, Del Bono V, Nicolini LA, Raiola AM, Gualandi F, Varaldo R, Di Muccio T, Gramiccia M, Gradoni L *et al*: Visceral leishmaniasis in hematopoietic cell transplantation: Case report and review of the literature. *Journal of Infection and Chemotherapy* 2018, 24(12):990-994. doi: 10.1016/j.jiac.2018.05.008.
35. Eichenberger A, Buechi AE, Neumayr A, Hatz C, Rauch A, Huguenot M, Diamantis-Karamitopoulou E, Staehelin C: A severe case of visceral leishmaniasis and liposomal amphotericin B treatment failure in an immunosuppressed patient 15 years after exposure. *BMC Infect Dis* 2017, 17(1):81. doi: 10.1186/s12879-017-2192-4.
36. Darcis G, Van der Auwera G, Giot J-B, Hayette M-P, Tassin F, Arrese Estrada J, Cnops L, Moutschen M, de Leval L, Leonard P: Recurrence of visceral and muco-cutaneous leishmaniasis in a patient under immunosuppressive therapy. *BMC Infectious Diseases* 2017, 17(1). doi: 10.1186/s12879-017-2571-x.
37. Prajapati R, Kumar A, Sharma P, Singla V, Bansal N, Dhawan S, Arora A: A Rare Presentation of Leishmaniasis. *Journal of Clinical and Experimental Hepatology* 2016, 6(2):146-148. doi: **https://doi.org/10.1016/j.jceh.2016.01.001**.
38. Chianura L, De Ferrari ME, Popescu CE, Cantoni S, Colussi G, Rossetti C, Puoti M: The role of F-18 FDG PET in the diagnosis of visceral leishmaniasis: two case reports. *Journal of Case Reports* 2014, 4(1):20-25. doi: 10.17659/01.2014.0006.
39. Comont T, Martin-Blondel G, Laurent C, Berry A, Marchou B: Severe haemophagocytic lymphohistiocytosis triggered by a visceral leishmaniasis in a patient with a Rosai-Dorfman disease. *Ann Hematol* 2014, 94(4):701-702. doi: 10.1007/s00277-014-2219-5.
40. Fuertes J, Garcia-Bennett JR, Iftimie S, Danús M, Abreu JA: Focal splenic FDG uptake in a patient with Kala-Azar (visceral leishmaniasis). *Clin Nucl Med* 2014, 39(4):387-390. doi: 10.1097/rlu.0000000000000369.
41. Valentina Gallina 1 RB, Arber Golemi 3, Mohsen Farsad 3, Günter Weiss 4, Christian J Wiedermann: Imported visceral leishmaniasis – unexpected bone marrow diagnosis in a patient with fever, pancytopenia, and splenomegaly. *Am J Blood Res* 2014, 4(2):101-105.
42. Guard D, Nigam S: 23. Case study: Visceral leishmaniasis - A rare cause of hepatosplenomegaly in australia. *Pathology* 2014, 46(S1):S114. doi: 10.1097/01.Pat.0000443713.70758.73.
43. Mao G, Yang G, Cheng Y, Zee CS, Huang W, Ni W, Meng G, Chen Z: Multiple Nodular lesions In Spleen Associated With Visceral Leishmaniasis. *Medicine* 2014, 93(29). doi: 10.1097/md.0000000000000272.
44. D.H. van Raalte HMW, G. de Klerk: Unexpected diagnosis of visceral leishmaniasis in a patient resenting with an infected ICD lead. *The Netherlands Journal of Medicine* 2014, 72(03):146-148.
45. Murri M, Fraticelli P, Goteri G, Mattioli M, Kafyeke A, Martino GP, Scortechini I, Leoni P, Gabrielli2 A: Visceral leishmaniasis in a patient with Coombs negative haemolytic anaemia and polycythemia vera:a case report. *Italian Journal of Medicine* 2014, 8(Suppl 2):89-89.
46. Gibson, G.M.; Arnold, C.; Kumar, A.S.R. 18F-FDG Uptake in Multiple Splenic Foci on PET/CT An Unusual Case of Visceral Leishmaniasis. *Clin. Nucl. Med.* 2014, 39, 828–830. doi: 10.1097/RLU.0000000000000378.
47. Evers G, Pohlen M, Berdel WE, Thoennissen NH, Titze U, Köhler G, Weckesser M, Anthoni C, Mesters RM: Visceral leishmaniasis clinically mimicking lymphoma. *Annals of Hematology* 2013, 93(5):885-887. doi: 10.1007/s00277-013-1896-9.

48. Berry CE, Tsai J, Tierney A, Pickles R: Visceral leishmaniasis in a patient taking adalimumab for rheumatoid arthritis. *Medical Journal of Australia* 2013, 198(6):331-333. doi: 10.5694/mja12.11003.
49. Wu L, Wang Y, Cui H, Wang Z: Hemophagocytic lymphohistiocytosis induced by Leishmania infection *Journal of Interna l Medicine Concepts & Practice* 2013, 8(03):213-214. doi: 10.3969/j.issn.1673-6087.2013.03.014.
50. Kyrtatos PG, Debard A, Martin-Blondel G, Alvarez M, Delobel P, Marchou B, Massip P, Wagner T: FDG-PET/CT findin gs during immune reconstitution in an HIV-1 patient infected with visceral leishmaniasis. *Infection* 2013, 41(5):1017-1019. doi: 10.1007/s15010-013-0459-2.
51. Claudia Ravaglia CG, 1 Gian Luca Casoni,1 Silvia Asioli,2 Venerino Poletti1: Fever, splenomegaly and lymphopenia in s arcoidosis. *Thorax* 2013, 68:496-497. doi: 10.1136/thoraxjnl-2011-201408.
52. Martin C, Castaigne C, Tondeur M, Flamen P, De Wit SJHm: Role and interpretation of fluorodeoxyglucose-positron em ission tomography/computed tomography in HIV-infected patients with fever of unknown origin: a prospective study. *HIV Medicine* 2013, 14(8):455-462. doi: 10.1111/hiv.12030.
53. Ali Fuat Yapar M, Mehmet Reyhan M, Nazim Emrah Kocer M, Mehmet Aydin M, Gu¨l Nihal Nursal M: Diffuse Spleni c F-18 FDG Uptake in Visceral Leishmaniasis. *Clinical Nuclear Medicine* 2011, 36(11):1041-1043. doi: 10.1097/RLU.0b013e31 821a2965.
54. Bielory BP, Lari HB, Mirani N, Kapila R, Fitzhugh VA, Turbin RE: Conjunctival Squamous Cell Carcinoma Harboring L eishmania Amastigotes in a Human Immunodeficiency Virus–Positive Patient. *Archives of Ophthalmology* 2011, 129(9):1230 -1231. doi: 10.1001/archophthalmol.2011.252.
55. Casadevall M, Mambré L, Cornet M, Caillat-Vigneron N, Le Jeunne C, Aslangul E: Contribution of 18-FDG positron em ission tomography for the diagnosis of visceral leishmaniasis. *Médecine et Maladies Infectieuses* 2010, 40(12):719-721. doi: 1 0.1016/j.medmal.2010.06.003.
56. Lupi A, Todeschini G, Zanco P: Diffuse metabolic activation of reticuloendothelium on F-18 FDG PET imaging in a cas e of visceral leishmania. *Clinical Nuclear Medicine* 2006, 31(1):34-36. doi: 10.1097/01.rlu.0000191574.30071.d7.
57. Ea V, Papa B, Goldberg R: A case of visceral leishmaniasis masquerading as autoimmune hepatitis. *Medical Journal of A ustralia* 2024, 221(6):299-301. doi: 10.5694/mja2.52412.
58. Pan Y, Kang H, Liu G, Liu Y, Li M, Wang C: Report of 3 misdiagnosed cases of kala-azar. *Academic Journal of Chinese P LA Medical School* 2014, 35(06):627-629. doi: 10.3969/j.issn.2095-5227.2014.06.031.
59. Fan F, Su Y, Wang Y, Liu Y, Yi H, Fu L, Qiu L: Kala-azar with hemophagocytic syndrome as the initial manifestation: a case report. *Journal of Third Military Medical University* 2012, 34(13):1335-1339. doi: 10.16016/j.1000-5404.2012.13.014.
60. Ibad IK, Vankayala A, Manwatkar A, Mathew J: Visceral leishmaniasis mimicking inflammatory myositis that led to hae mophagocytic lymphohistiocytosis. *BMJ Case Rep* 2025, 18(2):e259777.. doi: 10.1136/bcr-2024-259777.
61. Wang H, Li J, Huang W: Case Report: Fever of unknown origin with hemophagocytic lymphohistiocytosis and intestina l hemorrhage—a successfully treated case of severe visceral leishmaniasis. *Frontiers in Medicine* 2025, 12. doi: 10.3389/fme d.2025.1663551.
62. Tolaj I, Mehmeti M, Gashi H, Berisha F, Gashi V, Fejza H, Shala N: Visceral leishmaniasis in Kosovo: A case of misdia gnosis and diagnostic challenges. *IDCases* 2023, 32. doi: 10.1016/j.idcr.2023.e01768.
63. Liu Y, Guo Z, Sun L, Zhang D: Clinical and epidemiological investigation and analysis of a new visceral leishmaniasis case in Xin'an County, Henan Province. *Disease Surveillance* 2023, 38(09):1134-1137. doi: 10.3784/jbjc.202301200007.
64. Huang S, Zhao J, Li H, Chen A, Zhang X, Tang R, Yang R: Adult kala-azar complicated by multiple organ dysfunction syndrome: report of 3 cases *Jiangsu Medical Journal* 2023, 49(05):538-541. doi: 10.19460/j.cnki.0253-3685.2023.05.025.
65. Zhang G, Zhong J, Wang T, Zhong L: A case of visceral leishmaniasis found by left oblique hernia: A case report. *Exp Ther Med* 2020, 19(4):2697-2701. doi: 10.3892/etm.2020.8487.
66. Feifei L, Rugui L, Fang L: Clinical analysis of a couple with kala-azar complicated by splenic infarction. *Chinese Journal of Clinical Infectious Diseases 2017* 2017, 10(2):135-138. doi: 10.3760/cma.j.issn.1674-2397.2017.02.010.
67. Wu Y, Meng J, Zhang X, Chen J, Luan C, Tang G, He Z: Hemophagocytic syndrome induced by kala-azar: a case repo rt and literature review *Journal of Clinical Hematology* 2017, 30(01):71-72. doi: 10.13201/j.issn.1004-2806.2017.01.020.
68. Molinaro F, Varriale A, De Matthaeis A, De Cata A, Scalzulli P: Leishmaniasis: A misunderstood case of FUO. *Italian Jo urnal of Medicine* 2014, 8:88. doi: 10.4081/itjm.2014.s2.
69. Vase MO, Hellberg YK, Larsen CS, Petersen E, Schaumburg H, Bendix K, Ravel C, Bastien P, Christensen M, D'Amore F: Development of splenic marginal zone lymphoma in a HIV-negative patient with visceral leishmaniasis. *Acta Haemato logica* 2012, 128(1):20-22. doi: 10.1159/000337341.
70. Centers for Disease Control and Prevention; Myles, O.; Wortmann, G.; Barthel, R.; Ockenhouse, C. Two Cases of Viscer al Leishmaniasis in U.S. Military Personnel—Afghanistan, 2002–2004. *Morb. Mortal. Wkly. Rep.* **2004**, *53*, 265–268.
